# The risk of bone fracture after long-term risperidone exposure is not increased compared to other atypical antipsychotics: A retrospective cohort study

**DOI:** 10.1371/journal.pone.0221948

**Published:** 2019-09-05

**Authors:** Shih-Pei Shen, Yanfang Liu, Hong Qiu, Kuan-Yi Tsai, Hung-Chi Wu, Wen-Miin Liang, Meng Shu, Frank Huang-Chih Chou

**Affiliations:** 1 Department of Public Health, China Medical University, Taichung City, Taiwan; 2 Janssen Research & Development, LLC, Global Epidemiology, Titusville, New Jersey, United States of America; 3 Department of Community Psychiatry, Kai-Syuan Psychiatric Hospital, Kaohsiung City, Taiwan; 4 Department of Health Services Administration, China Medical University, Taichung City, Taiwan; 5 Janssen China R & D Center, Shanghai, China; Chiba Daigaku, JAPAN

## Abstract

**Objective:**

Antipsychotic agents can increase circulating serum prolactin levels, potentially leading to osteoporosis and increased risk of bone fracture. The risk appears to be lower for atypical antipsychotics. We investigated whether risperidone was associated with an increased fracture risk by estimating the incidence of hip/femur and non-hip/femur fractures in users of risperidone, other atypical, and typical antipsychotics.

**Methods:**

This retrospective cohort study with a nested case-control study used claims data from the Taiwan National Healthcare Insurance database. All new users of antipsychotics between 2000–2012 were included. Incident fractures were identified using ICD-9 codes from inpatient records. Cox proportional hazards models compared fracture incidence among exposure groups. Conditional logistic regression models compared antipsychotic exposure among fracture cases versus matched controls.

**Results:**

340,948 patients were included in the analysis. There were 2832 hip/femur fractures and 2693 non-hip/femur fractures: Hip/femur fracture incidence 636.8/100,000 person-years (Risperidone), 885.7/100,000 person-years (Other Atypical), 519.4/100,000 person-years (Typical). The adjusted hazard ratio of hip/femur fracture was 0.92 (95%CI 0.84–1.01) comparing Other Atypical with Risperidone, and 1.00 (95%CI 0.89–1.11) comparing Typical with Risperidone. The adjusted hazard ratio of non-hip/femur fracture was 1.08 (95%CI 0.98–1.20) for Other Atypical versus Risperidone, and 1.10 (95%CI 0.99–1.22) for Typical versus Risperidone. The adjusted odds ratio for hip/femur fractures was 0.92 (95% CI 0.83–1.01) in cases and controls exposed to other atypical antipsychotics compared with risperidone for 1 year prior to fracture date, 0.97 (95% CI 0.87–1.07) during 1–3 years, and 0.92 (95% CI 0.81–1.06) during 3–5 years prior to fracture date. The adjusted odds ratio for non-hip/femur fractures were 1.11 (95% CI 0.99–1.24), 1.02 (95% CI 0.0.91–1.14), and 0.95 (95% CI 0.82–1.09), respectively.

**Conclusion:**

There was no increased risk of bone fracture in long-term users of risperidone compared to users of other atypical antipsychotics.

## Introduction

Antipsychotic drugs exert variable antagonistic effects on the dopamine type 2 and serotonin type 2A receptors, which raises circulating prolactin levels [1, 2]. Hyperprolactinemia is associated with menstrual irregularities, galactorrhea, gynecomastia, reduced libido, sexual dysfunction, infertility, as well as decreased bone mineral density that may lead to an increased risk of fracture [2–4]. Persons with psychotic illnesses such as schizophrenia have an increased risk of osteoporosis and osteoporotic fracture compared with the general population, whether treated or untreated with antipsychotics [5–8]. The numerous factors that contribute to this increased risk include metabolic syndrome associated with the underlying disease, co-morbidities, lifestyle factors such as lack of physical activity, reduced exposure to sunlight, smoking, undernutrition, excessive alcohol consumption, and the side-effects of treatment. In early 2017, the United States Food and Drug Administration approved a labelling update for all antipsychotic medications, adding a new warning to the prescribing information stating that antipsychotic drugs may cause somnolence, postural hypotension, motor and sensory instability, which may lead to falls causing fractures or other injuries [9].

It is not currently known whether hyperprolactinemia induced by antipsychotics contributes directly to an increased risk of osteoporotic fracture [8]. Epidemiologic studies of the association between antipsychotics and the incidence of hip fracture have produced inconsistent findings. In studies that adjusted for schizophrenia diagnosis, some [10–13], but not all [14] studies found an independent association between antipsychotic drug use and hip fracture compared with non-use of antipsychotics. However, comparisons of antipsychotic drug users with non-users may be confounded by the underlying diseases [15].

Risperidone is an antipsychotic agent that belongs to the therapeutic drug class of atypical antipsychotics. Risperidone is associated with higher and more frequent elevations of circulating prolactin levels than other atypical antipsychotics [16, 17]. As a result, there has been concern that risperidone may be associated with an increased risk of osteoporosis-related fractures. The most recent-meta-analysis of fracture risk associated with anti-psychotic use included 36 observational studies and evaluated 448,368fracture cases. The analysis concluded that the risk of hip fracture increased significantly by 77% in users of first-generation antipsychotics compared to non-users, and by 41% in users of second-generation antipsychotics [18]. This study also noted differential risks associated with individual drug classes; risperidone was associated with a 29% increase in fracture risk, versus 46% for olanzapine, 49% for quetiapine and 94% for haloperidol, with the only statistical difference between risperidone and haloperidol.

Risperidone was the first atypical antipsychotic licensed for the treatment of schizophrenia in Taiwan. Few studies of fracture risk on patients taking anti-psychotics have been conducted in Asian populations, and these have been limited to hospitalized patients or to patients with schizophrenia [19, 20]. Furthermore, few prospective studies have evaluated the risk of fracture in long term users of antipsychotics, and available studies are limited by collection of data at baseline without information of ongoing or altered usage over the follow-up period [21–23]. To bridge this data gap we conducted a retrospective cohort study to compare the risk of osteoporosis-related fractures associated with risperidone versus other antipsychotic agents. A nested case-control study was used to complement the cohort study.

## Methods

### Study objectives and design

The primary study objective was to compare the incidence of hip/femur fractures in users of risperidone, users of other atypical antipsychotics, and users of typical antipsychotics. The secondary objective was to compare the incidence of non-hip/femur fractures in the different exposure groups.

#### Cohort study

**Fig 1:**
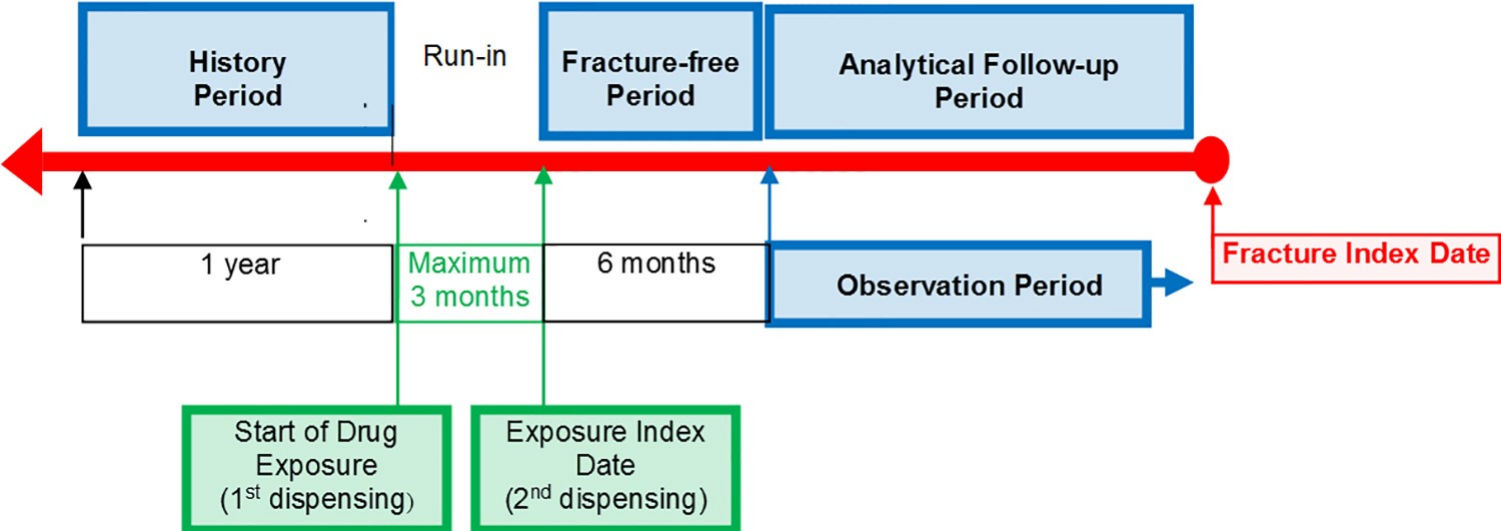
Study design. Patient follow-up ended at discontinuation (a gap of > 60 days) of the treatment regimen plus 6 months, occurrence of an osteoporosis-related fracture (defined as non-open fractures that occurred in the absence of major traumas or bone metastases), disenrollment from the database, or the end of study period (30 June 2012), whichever came first.

Patients newly exposed to an antipsychotic were identified in Taiwan’s National Health Insurance Research Database (NHIRD) and followed retrospectively for ascertainment of any new occurrences of hip/femur or non-hip/femur fractures during the study period. Exposure status was based on the earliest pair of consecutive prescriptions. The date of the second dispensing among the earliest pair of consecutive prescriptions in the databases was set as the exposure index date (Fig 1). Exposed individuals had to be new users of the antipsychotic, defined as having no prescription record for any antipsychotic in the 12 months prior to the first dispensing that defined the exposure group.

#### Nested case-control study

The nested case-control study evaluated the association between hip/femur fracture and current, recent and past exposure to risperidone compared with other atypical antipsychotics. Cases were patients with a newly diagnosed osteoporotic fracture. Previous bone fractures prior to the index date were excluded. For each fracture case, four controls were randomly selected from the new users of atypical antipsychotics on the date of the outcome event. Controls were matched by age (within a ± 4-year window), gender, and duration of drug exposure (± 60 days). Controls were assigned the fracture date of the case to which they were matched. For each case and control, drug exposure history in the 5 years prior to the osteoporosis-related fracture index date were divided into current, recent, and past periods. The current period was defined as within 1 year prior to the fracture index date, the recent period was between 1 year and 3 years, and the past period was between 3 years and 5 years prior to the fracture index date.

### Data source: Taiwan’s National Health Insurance Research Database (NHIRD)

We used data collected in the NHIRD from 01 June 2000 through 30 June 2012. The NHIRD is a large, representative, population-based database provided by the Taiwan National Health Research Institute using claims data from the National Health Insurance program. The National Health Insurance program provides mandatory universal health insurance for approximately 99% of >23 million people in Taiwan and was implemented in March 1995. The NHIRD holds a comprehensive set of patient and clinical information, including demographic data, International Classification of Diseases– 9^th^ revision (ICD-9) diagnostic codes, dates and types of procedures, dispensed prescription drugs, and expenditures. Quality of the dataset is monitored by the National Health Insurance Bureau of Taiwan, which randomly reviews the charts of one per 100 ambulatory, and one per 20 inpatient claim cases, and interviews patients to verify the accuracy of the diagnosis [24–26].

A separate subset of the NHIRD is the Registry of Catastrophic Illness (NHIRD-RCI). Insured patients who suffer from certain major diseases can apply for a catastrophic illness certificate which grants exemption from all co-payments related to that disorder. For example, schizophrenia is classified as a major catastrophic illness once the diagnosis has been verified. NHIRD-RCI data were also used to identify major injury and malignancy that would lead to exclusion of a fracture case.

### Study population

Individuals enrolled in the NHIRD with exposure to an antipsychotic between 01 June 2001 through 30 June 2012 were identified by the presence of an antipsychotic prescription/dispensing record in the database, classified as risperidone, any other atypical antipsychotic (except paliperidone), or conventional (typical) antipsychotic using the Anatomical Therapeutic Chemical classification (Supplementary material).

Eligible study subjects were at least 18 years of age at the exposure index date, had been enrolled in the database for at least 12 months prior to the first identified antipsychotic medication, and had at least two consecutive dispensings of an antipsychotic during the study period (Fig 1).

Patients with hip/femur fracture prior to the exposure index date or within 6 months after the exposure index date were excluded from the study population. Patients were also excluded if they had active cancer or an ICD code for a pituitary tumor at the exposure index date or within 5 years previously; if they had less than 6 months of follow-up after index exposure; or if paliperidone had been dispensed at any time. Individuals were also excluded if their fracture diagnosis was linked to an ICD-9 code indicating major trauma. Other baseline information was collected for a 12-month period prior to exposure.

### Outcomes

The primary study outcome was occurrence of incident hip/femur fractures identified from inpatient claims ICD diagnostic codes, in patient and who had hip/femur surgical procedures and/or X-ray within 4 weeks of the diagnosis.

Incident non-hip/femur fractures (secondary outcome) were identified both inpatient (including emergency room) and outpatient records, based on ICD diagnostic codes. Because fracture rates at other anatomical sites were low, fractures of the spine, clavicle, ribs, wrist, humerus, radius/ulna, pelvis, and tibia/fibula were grouped together (non-hip/femur fractures).

### Potential confounders

For adjustment purposes, the prevalence of comorbidities such as psychiatric conditions and somatic disease associated with elevated levels of prolactin was investigated based on previously recorded diagnoses in the national patient register beginning 6 months prior to the exposure index date. Data on concomitant psychiatric medication and medication treatments for hyperprolactinemia and non-antipsychotic drugs associated with increased prolactin levels were also collected from the NHIRD beginning 6 months prior to the exposure index date. Any previous psychiatric diagnoses recorded within the study observational period prior to the first of the two consecutive dispensations were considered as potential confounders.

### Statistical analysis

Data management and analyses were conducted using SAS software, Version 9.4 (SAS Institute Inc., Cary, NC, USA).

#### Sample size estimation

It was estimated that 13,000 individuals newly exposed to risperidone would be needed to detect a relative risk of 2.0 or greater with 85% power, and that 39,000 patients newly exposed to risperidone would be needed to detect a relative risk of 1.5 or greater with 80% power, assuming a background incidence rate for hip/femur fractures of 2 per 1,000 person-years in Taiwan [27]. For the nested case-control analysis, at least 240 cases eligible for study inclusion would be needed among the study population of new users of any atypical antipsychotic (including risperidone) to detect an odds ratio of 1.5 or greater with 80% power. With 90 cases, the study would have 82% power to detect an odds ratio of 2.0 or greater.

#### Cohort study

Fracture incidence rates were calculated according to the person-time of treatment follow-up. Incidence rates in the Other Atypical and Typical groups were compared with rates in the Risperidone group using Cox proportional hazards regression models. Hazard ratios (HRs) and their 95% confidence intervals (CIs) were estimated. The time-to-first-event was modelled in this analysis. Covariates were only retained in the final Cox regression model if their inclusion in a model containing the single covariate and the antipsychotic exposure variable changed the HR for the antipsychotic exposure variable by 10% or more, relative to the unadjusted HR for antipsychotic exposure (i.e., adjusted HR/unadjusted HR is either >1.10 or <0.90) [28].

#### Case-control study

The following patients were excluded from the analysis for the primary model: (1) those with polypharmacy (overlapping prescriptions of risperidone and other atypical antipsychotics > 60 days); (2) those who switched prescriptions from risperidone to other atypical antipsychotics (a change of antipsychotic medications with an overlap < 60 days or gap ≤ 60 days); and (3) those with a total duration of antipsychotic drug exposure of less than 28 days. The proportions of excluded individuals in the exposed and unexposed cohorts were checked to ensure there were no major differences between the groups.

To evaluate robustness of results for time dependent antipsychotic exposures, conditional logistic regression models were used to estimate odds ratios (ORs) and their 95% CIs of an osteoporosis-related fracture diagnosis according to antipsychotic exposure among cases versus controls. Potential confounding variables were selected by information yielded in the cohort analysis. A *post hoc* analysis estimated ORs for the hip/femur and non-hip/femur fracture risk among users of individual atypical antipsychotics (aripiprazole, clozapine, olanzapine, quetiapine, ziprasidone).

### Ethics approval

The study was approved by the Ethics Review Board of Kaohsiung Municipal Kai-Syuan Psychiatric Hospital (approval number KSPH-2015-09). All personally identifiable information was encrypted to protect patient privacy.

## Results

### Characteristics of study subjects

Between 2001 and 31 June 2012 (2000–2001 was a baseline period) there were 148,699 individuals exposed to risperidone, 258,957 individuals exposed to other atypical antipsychotics, and 341,073 individuals exposed to typical antipsychotics in the NHIRD (S1 Fig). After exclusion criteria were applied, 73,315, 120,538 and 147,095 male and female patients remained in the Risperidone, Other Atypical and Typical groups, respectively.

The exposure groups were similar with respect to age and gender. Across the study groups, the percentage of patients who were men ranged between 48.0% and 52.6%, and the mean age at inclusion ranged from 50.3 to 54.8 years (Table 1). The proportion of patients who were over 80 years of age at the time of the first recorded antipsychotic prescription was highest in the Other Atypical group (15.9%), and lowest (7.38%) in the Typical group. Each year, the number of patients in the database decreased in the Risperidone and Typical groups, whereas the number of patients in the Other Atypical group increased.

**Table 1:** Baseline characteristics of patients exposed to risperidone, other atypical antipsychotics and typical antipsychotics.

Total number included	Risperidone N = 73,315		Other Atypical N = 120,538		Typical N = 147,095	
	number	%	number	%	number	%
**Gender**						
Male	38,549	52.6	57,793	48.0	75,328	51.2
Female	34,766	47.4	62,745	52.1	71,767	48.8
**Age at inclusion (years)**						
Mean (SD)	51.25	(21.5)	54.77	(21.2)	50.30	(19.0)
**Age group**						
18–39	27,130	37.0	36,126	30.0	48,629	33.1
40–49	10,365	14.1	17,421	14.5	28,285	19.2
50–59	8349	11.4	14,796	12.3	23,074	15.7
60–69	7170	9.8	12,984	10.8	17,804	12.1
70–79	10,974	15.0	20,045	16.0	18,447	12.5
≥80	9327	12.7	19,166	15.9	10,856	7.4
**Index year**						
2001	7395	10.1	3298	2.7	25,062	17.0
2002	7002	9.6	4464	3.7	19,930	13.6
2003	8125	11.1	7044	5.8	16,590	11.3
2004	7824	10.7	8658	7.2	15,421	10.5
2005	6647	9.1	9523	7.9	12,983	8.8
2006	6363	8.7	9982	8.3	10,175	6.9
2007	6548	8.9	10,420	8.6	8690	5.9
2008	5904	8.1	12,074	10.0	8020	5.5
2009	5141	7.0	13,606	11.3	9264	6.3
2010	5011	6.8	15,862	13.2	8588	5.8
2011	4781	6.5	16,542	13.7	8458	5.8
2012.1–2012.6^$^	2574	3.5	9065	7.5	3914	2.7
**Primary indication for major psychiatric diseases**						
Schizophrenia	22,926	31.3	13,643	11.3	18,689	12.7
Bipolar disorder	4350	5.9	11,581	9.6	5597	3.8
Major depression	5092	7.0	18,026	15.0	5922	4.0
Dementia	12,723	17.4	21,159	17.6	5607	3.8
Others psychiatric diseases or behavior (including: Autism & Disruptive behaviors)	75	0.10	49	0.04	61	0.04
**2000–2012 diagnosis for major psychiatric diseases**						
Schizophrenia	29,388	40.1	19,537	16.2	26,567	18.1
Bipolar disorder	9076	12.4	21,243	17.6	14,005	9.5
Major depression	12,137	16.6	33,954	28.2	19,288	13.1
Dementia	18,890	25.8	31,848	26.4	15,718	10.7
Others psychiatric diseases or behavior (including: Autism & Disruptive behaviors)	619	0.84	433	0.36	523	0.36

^$^ Year 2012 only included subjects for half a year (from 2012.1 to 2012.6)

N = total number included, SD = standard deviation

The percentage of patients who had a primary indication for a major psychiatric disease was 61.6% in the Risperidone group, 53.5% in the Other Atypical group and 24.4% in the Typical group. The percentage of patients with a primary indication for schizophrenia was 31.3% in the Risperidone group, versus 11.3% and 12.7% in the Other Atypical and Typical groups, respectively. The percentage of patients who had any diagnosis of a major psychiatric disease over the study period was 95.6% in the Risperidone group, 88.8% in the Other Atypical group and 51.7% in the Typical group. The most common primary indications for antipsychotic use were schizophrenia (31.3%) and dementia (17.4%) in the Risperidone group, dementia (17.6%) and major depression (15.0%) in the Other Atypical group, and schizophrenia (12.7%) and major depression (4.0%) in the Typical group.

Table 2 shows psychiatric and somatic medical history and previous medication use relevant to bone integrity for 12 months prior to the index exposure. The most common diagnosis in all groups was Neurotic stress-related or somatoform disorder (16.5% to 29.9% of patients across groups), followed by schizophrenia in the Risperidone and Typical groups (12.9% and 6.0% of patients, respectively), and major depression in the Other Atypical group (12.2% of patients). There were high rates of use of anxiolytics, sedatives and hypnotics (46.5% to 65.5% of patients across groups), as well as anti-depressants (17.5% to 37.5% of patients across groups) 12 months before the index exposure date. Between 8.8% to 12.1% of patients in each group also received opiates during this period. The highest users of anxiolytics, sedatives and hypnotics, antidepressants and opiates were patients in the Other Atypical group.

**Table 2:** Medical history and use of medication prior to the index date.

	Risperidone N = 73,315		Other Atypical N = 120,538		Typical N = 147,095		P-value
	number	%	number	%	number	%	
**Inpatient and outpatient diagnosis 12 months prior to the index exposure**							
Schizophrenia	9436	12.9	6379	5.3	8830	6.0	<0.0001
Bipolar disorder	1938	2.6	4651	3.9	2878	2.0	<0.0001
Major depression	4160	5.7	14678	12.2	6299	4.3	<0.0001
Dementia	4631	6.3	8490	7.0	3209	2.2	<0.0001
Seizures/Epilepsies	1555	2.1	1994	1.7	2264	1.5	<0.0001
Renal dysfunction	1318	1.8	3370	2.8	2711	1.8	<0.0001
Other organic psychiatric disorder	2152	2.9	3163	2.6	2139	1.5	<0.0001
Other psychosis	2347	3.2	2272	1.9	1764	1.2	<0.0001
Neurotic stress related or somatoform disorder	12,125	16.5	36,064	29.9	25,200	17.1	<0.0001
Alcohol or other substance use disorder	790	1.1	2069	1.7	2273	1.6	<0.0001
Other psychiatric diseases	1186	1.6	1656	1.4	1220	0.83	<0.0001
Other physical condition	28	0.04	72	0.06	61	0.04	0.04
**Inpatient and outpatient possible bone-related medications 12 months prior to the index exposure**							
Glucocorticosteroids	3512	4.8	7987	6.6	9314	6.3	<0.0001
Anxiolytics, sedatives and hypnotics	36,538	49.8	78993	65.5	68319	46.5	<0.0001
Antidepressants	16,226	22.1	45,166	37.5	25,704	17.5	<0.0001
Proton-pump inhibitors	2733	3.7	8647	7.2	7039	4.8	<0.0001
Hormone replacement therapy	853	1.2	1741	1.4	2748	1.9	<0.0001
Oral contraceptives	1169	1.6	2958	2.5	4169	2.8	<0.0001
Calcium supplements	2013	2.8	2658	2.2	5670	3.9	<0.0001
Thiazide diuretics	3634	5.0	8020	6.7	6496	4.4	<0.0001
Gastrointestinal medications	7047	9.6	16,975	14.1	19,239	13.1	<0.0001
Opiates	6458	8.8	14,608	12.1	13,552	9.2	<0.0001
Antihypertensive medications	1240	1.7	2116	1.8	3374	2.3	<0.0001
Glucorticosteroids ≥ 90 days	1086	1.5	2720	2.3	3098	2.1	<0.0001
Others	158	0.22	608	0.50	321	0.22	<0.0001

N = total number included.

### Incidence of hip/femur fractures

Approximately 85% of hip/femur fractures were identified from the inpatient file. There were 730 incident osteoporosis-related hip/femur fracture cases identified from inpatients in the Risperidone group. All cases were confirmed as having hip/femur fracture procedures and/or X ray within 4 weeks of the fracture diagnosis. There were 1379 cases identified in the Other Atypical group and 723 in the Typical group. The mean duration of follow-up was 1.56 years in the Risperidone group, 1.29 years in the Other Atypical group and 0.95 years in the Typical group (Table A in S1 File). The crude incidence of hip/femur fracture was 636.78 per 100,000 person-years in the Risperidone group, 885.67 per 100,000 person-years in the Other Atypical group and 519.35 per 100,000 person-years in the Typical group (Table 3).

**Table 3:** Results of the cohort analysis of fractures using Cox-regression model.

	PYR	Number of cases	Cases/ 100,000 PYR	Crude HR	Crude 95% CI	Adjusted^$^ HR	Adjusted^$^ 95% CI
**Hip/femur fractures**^%^							
Risperidone	114640	730	636.78	Ref = 1	-	Ref = 1	-
Other atypical	155701	1379	885.67	1.33	(1.22–1.46)	0.92	(0.84–1.01)
Typical	139213	723	519.35	0.78	(0.71–0.87)	0.996	(0.89–1.11)
Age group							
18–39	144822	140	96.67	Ref = 1	-	Ref = 1	-
40–49	76488	125	163.42	1.7	(1.33–2.16)	1.69	(1.33–2.15)
50–59	55678	157	281.98	2.96	(2.35–3.71)	2.86	(2.28–3.6)
60–69	42674	353	827.20	8.72	(7.17–10.61)	7.61	(6.2–9.33)
70–79	53149	973	1830.70	19.46	(16.28–23.25)	15.18	(12.45–18.5)
≥80	36742	1084	2950.30	31.56	(26.41–37.7)	23.42	(19.13–28.67)
Index diagnosis of schizophrenia	123068	268	217.77	0.25	(0.22–0.29)	0.84	(0.67–1.05)
Index diagnosis of dementia	44434	1074	2417.07	4.88	(4.52–5.26)	1.12	(1.01–1.23)
Any time diagnosis of schizophrenia	159688	398	249.24	0.26	(0.23–0.29)	1.11	(0.92–1.32)
Any time diagnosis of dementia	81170	1735	2137.49	6.29	(5.83–6.79)	1.39	(1.25–1.55)
One year before exposure date diagnosed with schizophrenia	67675	155	229.04	0.31	(0.26–0.37)	1.06	(0.85–1.32)
**Non-hip/femur fractures**							
Risperidone	114504	646	564.2	Ref = 1	-	Ref = 1	-
Other atypical	155847	1092	700.7	1.22	(1.1–1.34)	1.08	(0.98–1.2)
Typical	138576	955	689.2	1.2	(1.08–1.32)	1.1	(0.99–1.22)
Index diagnosis of schizophrenia	122319	577	471.7	0.66	(0.60–0.72)	0.69	(0.60–0.80)
Any time diagnosis of schizophrenia	158709	831	523.6	0.72	(0.66–0.79)	0.96	(0.84–1.09)

^$^ Adjusted variables including antipsychotic drug, age-group, Index and any time diagnosis (Schizophrenia, dementia), and one year before exposure date diagnosed as schizophrenia for hip/femur fracture and antipsychotic drug, index and any time diagnosis schizophrenia for non-hip/femur fractures.

^**%**^inpatient cases with hip/femur fracture procedure and X-ray within 4 weeks of the diagnosis, PYR = person-years, CI = 95% confidence interval, HR = hazard ratio

We conducted sensitivity analyses using different outcome definitions for hip/femur fractures (inpatient claim only and inpatient and/or outpatient claim). The results of the sensitivity analyses showed no significant difference in the risk of hip/femur fractures either in Other Atypical or Typical groups compared with the Risperidone group using any of the definitions of hip/femur fracture (Tables B and C in S1 File).

### Incidence of non-hip/femur fractures

The number of incident non-hip/femur fractures diagnosed from inpatient or outpatient records was 646 in the Risperidone group, 1092 in the Other Atypical group, and 955 in the Typical group (Table A in S1 File). The crude incidence of hip/femur fracture was 564.2 per 100,000 person-years in the Risperidone group, 700.7 per 100,000 person-years in the Other Atypical group and 689.2 per 100,000 person-years in the Typical group (Table 3).

### Cohort study: Associations between antipsychotic use and osteoporosis-related hip/femur fracture

Associations between the use of antipsychotic agents and osteoporosis-related hip/femur fractures using the Cox-regression model were reported as crude (unadjusted) HRs and adjusted HRs (aHR). There was no significant difference in the risk of hip/femur fractures in either the Other Atypical or Typical groups compared with the Risperidone group as the reference group (Table 3). The aHR was 0.92 (95% CI 0.84–1.01) for the Other Atypical group compared to the Risperidone group, and 0.996 (95% CI 0.89–1.11) for the Typical group compared to the Risperidone group. We observed that adjustment of the HR for the risk of hip/femur fractures in the Other Atypical compared with the Risperidone group resulted in a change in direction of the estimate (crude HR 1.33, 95% CI 1.22–1.46). Stratified analyses determined that age ≥80 years was the confounding factor that exerted the most effect on the results (Tables D to K in S1 File). Crude and aHRs for the risk of hip/femur fractures in the Other Atypical compared with the Risperidone group in patients aged ≥80 years were 0.91 (95% CI 0.79–1.05) and 0.93 (95% CI 0.8–1.08), respectively. There were more patients aged ≥80 years in the Other Atypical group than in the Risperidone group, leading to a reversal of the HR after adjustment.

There was no significant difference in the risk of non-hip/femur fractures in either the Other Atypical or Typical groups compared with the Risperidone group (Table 3). The aHR was 1.08 (95% CI 0.98–1.20) for the Other Atypical group compared to the Risperidone group, and 1.10 (95% CI 0.99–1.22) for the Typical group compared to the Risperidone group.

The risk of hip/femur fracture increased with age and was highest in those aged 80 years and over (aHR 23.52 95% CI 19.25–28.74) compared with the reference group of 18–39 year-olds (Table 3). The risk of hip/femur fracture was also increased in patients with an index diagnosis of dementia (aHR 1.11, 95% CI 1.01–1.22) and dementia diagnosed at any time (aHR 1.40, 95% CI 1.26–1.56). The risk of non-hip/femur fracture was decreased in patients with an index diagnosis of schizophrenia (aHR 0.69, 95% CI 0.6–0.8).

The confounding variables selected based on minimum of 10% change in the crude estimate after introducing the candidate covariate in the model of hip/femur fractures were antipsychotic drug, age-group, index-and any time diagnosis of schizophrenia or dementia, and one year before exposure date diagnosed with schizophrenia (Table L in S1 File). Confounding factors selected for the analysis of non-hip/femur fractures were antipsychotic drug, index-and any time diagnosis of schizophrenia (Table M in S1 File).

#### Nested-case control study: Antipsychotic exposure among fracture cases versus controls

The case-control study included 2535 patients with osteoporosis-related fractures of the hip and femur and 10,140 matched controls during the current period (within 12 months prior to the fracture index date), 2460 cases and 9,840 matched controls during the recent period (between 1–3 years prior to fracture index date), and 1,325 cases and 5,300 controls during the past period (between 3–5 years prior to facture index date). The groups were well matched with respect to age, gender, and duration of exposure. Characteristics of cases and controls are given in Table N in S1 File.

The associations between antipsychotic exposure and hip/femur and non-hip/femur fracture are reported as ORs and adjusted ORs (aOR) (Table 4). There was no difference in the risk of osteoporosis-related hip/femur fractures in patients exposed to other atypical antipsychotics compared with risperidone: aOR (0.92 (95% CI 0.83–1.01) for the current period, 0.97 (95% CI 0.87–1.07) for the recent period, and 0.92 (95% CI 0.81–1.06) for the past period. Similarly, there was no difference in the risk of osteoporosis-related non-hip/femur fractures in patients exposed to other atypical antipsychotics compared with risperidone: aOR 1.11 (95% CI 0.99–1.24) for the current period, 1.02 (95% CI 0.0.91–1.14) for the recent period, and 0.95 (95% CI 0.82–1.09) for the past period.

**Table 4:** Crude and adjusted odds ratio (OR) for antipsychotic exposure among cases versus controls.

	N of Cases	N of Controls	Crude OR (95% CI)	Adjusted OR$ (95% CI)
**Hip/femur fracture**				
*Current period*				
Risperidone	742	2892	1 (reference)	1 (reference)
Other Atypical	1639	6742	0.94 (0.85–1.04)	0.92 (0.83–1.01)
*Recent period*				
Risperidone	806	3083	1 (reference)	1 (reference)
Other Atypical	1535	5972	1.0 (0.90–1.10)	0.97 (0.87–1.07)
*Past period*				
Risperidone	493	1824	1 (reference)	1 (reference)
Other Atypical	697	2707	0.97 (0.85–1.11)	0.92 (0.81–1.06)
**Non-hip/femur fracture**				**Adjusted OR**^%^ **(95% CI)**
*Current period*				
Risperidone	613	2745	1 (reference)	1 (reference)
Other Atypical	1388	5289	1.19 (1.06 1.32)	1.11 (0.99–1.24)
*Recent period*				
Risperidone	670	2780	1 (reference)	1 (reference)
Other Atypical	1272	4788	1.12 (1.00–1.25)	1.02 (0.91–1.14)
*Past period*				
Risperidone	485	1952	1 (reference)	1 (reference)
Other Atypical	659	2502	1.08 (0.94–1.23)	0.95 (0.82–1.09)

^**$**^ Multivariate logistic regression model adjusting for age group, index and any time diagnosis (schizophrenia, dementia), and one year before exposure date diagnosed with schizophrenia

^%^ Multivariate logistic regression model adjusting for index and anytime schizophrenia diagnosis

Current period: within 1 year prior to the fracture index date,

Recent period: between 1 year and 3 years prior to the fracture index date,

Past period: between 3 years and 5 years prior to the fracture index date.

OR = odds ratio,

CI = confidence intervals,

N = number of patients

A *post hoc* analysis of fracture risk by individual atypical anti-psychotic found no difference in the risk of osteoporosis-related hip/femur fractures in patients exposed to aripiprazole, clozapine, olanzapine, quetiapine, or ziprasidone compared with risperidone (Table O in S1 File). There was a decreased risk of non-hip/femur fractures patients exposed to clozapine compared to risperidone across all three exposure periods, and an increased risk in patients exposed to olanzapine and quetiapine in the current period (Table P in S1 File). With the exception of quetiapine, the number of cases for each individual atypical antipsychotic was small (for example 27 to 47 for clozapine). In view of the low cases numbers and numerous statistical comparisons done without adjustment for multiplicity, these findings should be interpreted cautiously.

Crude and adjusted ORs were calculated for cases versus controls in the subset of patients who remained on the same antipsychotic drug throughout the recent and current periods, and throughout the past and current periods (Table Q in S1 File). In the adjusted analysis, there was no difference in the risk of osteoporosis-related hip/femur or non-hip/femur fractures in patients exposed to other atypical antipsychotics compared with risperidone in the sub-group of patients who took the same antipsychotic for up to 5 years.

## Discussion

We used a large, nationwide claims database containing comprehensive patient information over an 11-year period to estimate fracture risk according to antipsychotic use.

The results of the adjusted main analysis of hip/femur fracture as well as the secondary analysis of non-hip/femur fractures did not show any difference in fracture risk among users of risperidone and users of other atypical antipsychotics or typical antipsychotics. Age was a significant confounding factor, with the highest hip/femur fracture incidence observed in ≥80 year-olds. A diagnosis of dementia was also associated with an increased risk of hip/femur fracture, which may be linked to the association between older age and dementia onset.

The cause of osteoporotic fractures in patients taking antipsychotic drugs is multi-factorial and complex. As such, it is challenging to untangle the relative contributions of the underlying illness, co-morbidities, lifestyle choices, and antipsychotic treatments, on fracture risk. Although they provide insights into relationships between variables outcomes, observational study designs are unable to establish causal relationships between exposure and outcome. Observational studies may additionally be limited by inaccurate data, and inability for control for confounding by lifestyle factors, comorbidities and disease severity [29]. Study designs such as case-control have greater ability to account for confounding factors, but are unable to determing incidence rates or demonstrate temporal relationships. In the mixed retrospective cohort and case-control design used in our study, detailed analysis of the medical and drug history of patients allowed identification of potential cofounding variables related to comorbidity, drug and disease history, and their adjustment in the analysis. However, cannot exclude that other confounding variables as lifestyle factors (smoking, diet, physical activity, and underlying bone density) or other unknown factors may have influenced the results.

Age-group and underlying diagnoses of schizophrenia and dementia were identified as potentially confounding variables, which is not unexpected given that falls and fracture risk increase with age, are higher in patients with schizophrenia, and are higher in patients who are cognitively impaired [8, 30]. We noted high rates of ancillary drug use, particularly anxiolytics, sedatives and hypnotics but also anti-depressants and opiates in each of the exposure groups in the 12-month period before the index exposure date. Patients in the Other Atypical group were the highest users of these drugs and were also older and more likely to have a history of bipolar disorder or major depression and less likely to have schizophrenia than patients in the Risperidone and Typical exposure groups. Dementia was the most frequent primary drug indication in the Other Atypical group. The high rate of non-psychotic illness and ancillary drug use in the Other Atypical group reflects patterns of use of atypical antipsychotics in Taiwan, which are frequently used to treat non-psychotic illnesses including mood disorders, sleep disorders (as adjunctive treatment) and other physical conditions combined with sleep disturbance or psychiatric symptoms such as anxiety or depressed mood.

Risperidone has been marketed in Taiwan since 2000 and its use appeared to remain constant during the study. The use of other atypical antipsychotics increased steadily over the study period, especially from 2008, whereas the use of typical antipsychotic drugs that have been available for several decades, decreased markedly over the study period. Only 31.3% of patients who received Risperidone and 11.3% of those who received other atypical antipsychotics had a primary indication of schizophrenia, which could be explained by the off-label use of these medications for indications such as short-term sedation. Similar proportions were observed in a study of antipsychotic drug use in Sweden [31].

The Typical group had the longest total person-years of follow-up time but the shortest treatment follow-up duration. This is likely due to the higher percentage of patients in this group who had no major psychiatric diagnosis (48.26%), reflecting widespread use of sulpiride, an antipsychotic commonly used to treat gastrointestinal conditions in Taiwan. By contrast, the Risperidone group had the longest treatment follow-up time.

The mechanisms underlying the potential impacts of antipsychotic drugs on bone metabolism remain unclear. Antipsychotic-induced hyperprolactinemia is thought to inhibit the hypothalamo-pituitary-gonadal axis, with suppression of gonadal hormone levels leading to abnormal bone metabolism and osteoporosis [8, 32]. Prolactin may also exert a direct effect on osteoclasts, reducing cell proliferation and subsequently, osteoclast numbers in bone [8]. A prospective study of women with schizophrenia showed a direct association between raised prolactin levels and bone metabolism (formation and resorption), but no association with changes in bone mineral density of the femur or vertebrae, although the study follow-up period was limited to 12 months [33]. By contrast, evaluation of a biological marker of bone resorption (TRACP-5b) in patients with schizophrenia found lower bone metabolism compared to controls but noted marked differences in levels between men and women [34]. This study highlighted the complex relationships between sex hormones, prolactin levels and individual factor such as age and weight that potentially all impact bone metabolism in patients taking antipsychotics [34]. Strengths of the study were the use of the nationwide, population-based claims NHIRD. The large number of included patients and person-time of follow-up generated enough power to detect even a small change in the risk of developing an osteoporosis-related fracture. The nested case-control approach avoided the challenge of having to compare across exposures that varied over time using time-dependent variables (for exposure) in Cox models. It also simplified the challenges of adjusting for confounders that varied over time. By using real-world data, our results reflect the use of antipsychotics in clinical practice.

Potential limitations of the study were that information on the duration of psychiatric conditions, the average daily dose and the duration of the most recent course of antipsychotic therapy was not available in the NHIRD and unable to be included in the analysis. This means we were unable to detect possible antipsychotic dose-dependent impacts on fracture risk. However, this limitation was partially overcome by estimating fracture risk in patients who remained on the same antipsychotic for prolonged period (up to 5 years). The absence of any difference in fracture risk in patients who took risperidone or other atypical antipsychotic for up to 5 years, and who therefore received a high overall drug dose, argues against a dose-dependent effect. We divided antipsychotics into first generation drugs (typical antipsychotics) and second generation drugs (other atypical antipsychotics), and while drugs within these classes may vary in their potential association with fracture risk, the sample size of our study was not large enough to analyze fracture risk by individual drug exposure. Nevertheless, the results of our study are in accord with the conclusion of the meta-analysis reported by Papola et al, 2018 [18]. The NHIRD does not contain information about all potential covariates that could introduce confounding. While we selected all the available variables from the NHIRD, we cannot exclude an impact of confounding from other covariates. This is a potential limitation of all studies using the NHIRD.

As yet, the mechanisms by which antipsychotics affect bone metabolism remain unclear. The time needed for any such effect to result in an increased fracture risk is not known but is likely to differ according to the antipsychotic used, and to a range of other factors that may be genetic, phenotypic or behavioral in nature. Although observational studies have described links between antipsychotic use and fracture risk they are limited by their inability to make causal associations or to make links with biological parameters of bone metabolism. The availability of biological markers such as TRACP-5b [34], could be used to clarify the effect of antipsychotic drugs on bone metabolism. More precise mechanistic information could in turn, inform future study design aiming to make definitive connections between individual antipsychotics, treatment duration and fracture risk. In conclusion, the results of this large cohort study with a nested case-control study using real-world nationwide data from Taiwan showed no increased risk of bone fracture in long term users of risperidone compared to users of other atypical antipsychotic drugs.

## Supporting information

S1 File Drug ATC codes, sensitivity analyses, stratified analyses and *post hoc* analyses. (PDF)

S1 Fig Cohort creation flowchart. (TIF)
