# Supplementary material for: The risk of bone fracture after long-term risperidone exposure is not increased compared to other atypical antipsychotics: A retrospective cohort study
Source: PLoS One. 2019 Sep 5;14(9):e0221948. doi: 10.1371/journal.pone.0221948 (PMC6728018; doi:10.1371/journal.pone.0221948)
Supplement: S1 File — (PDF) [file pone.0221948.s001.pdf]

The Risk of Bone Fracture after Long-term Risperidone Exposure is Not Increased Compared to Other Atypical Antipsychotics: A Retrospective Cohort Study

Shih-Pei Shen[1], Yanfang Liu[2], Hong Qiu[2], Kuan-Yi Tsai[3], Hung-Chi Wu[3], Wen-Miin Liang[4], Meng Shu[5], Frank Huang-Chih Chou[3]

1. Department of Public Health, China Medical University, Taichung City, Taiwan

2. Janssen Research & Development, 920 Route 202, Raritan, NJ 08869, United States

3. Department of Community Psychiatry, Kai-Syuan Psychiatric Hospital, Kaohsiung City, Taiwan

4. Department of Health Services Administration, China Medical University, Taichung City, Taiwan

5. Janssen China R & D Center, Shanghai, China

Corresponding author: Dr. Frank Huang-Chih Chou, E-mail: f50911.tw@yahoo.com.tw

Supplementary Material

DRUG ATC CODES

A.1 Atypical Antipsychotics
aripiprazole N05AX12
clozapine N05AH02
olanzapine N05AH03
quetiapine N05AH04
risperidone N05AX08
ziprasidone N05AE04

A.2 Conventional Antipsychotics
Phenothiazines
chlorpromazine N05AA01
promazine N05AA03
fluphenazine N05AB02
perphenazine N05AB03
trifluoperazine N05AB06
thioridazine N05AC02
Butyrophenone derivatives
haloperidol N05AD01
Diphenylbutylepiperdine derivatives
pimozide N05AG02
Other
loxapine N05AH01

Table A. The incidence of fractures in patients exposed to risperidone, other atypical antipsychotics and typical antipsychotics

	Risperidone N=73,315	Other Atypical N=120,538	Typical N=147,095
**Hip/femur fractures (inpatient diagnosis)**			
PYR active treatment follow-up time			
Total	114,630	155,682	139,200
Mean (SD)	1.56 (2.35)	1.29 (1.84)	0.95 (1.72)
Number censored			
Death	10,292	13,305	16,181
Emigration	5234	6748	11,611
Number of fracture cases (%)	747 (1.0)	1400 (1.2)	733 (0.50)
Procedures or X-ray within 4 weeks of the hip/femur fracture diagnosis	730 (97.7)	1379 (98.5)	723 (98.6)
**Non-hip/femur fractures (inpatient and outpatient diagnosis)**			
PYR active treatment follow-up time (years)			
Total	114504	155847	138576
Mean (SD)	1.56 (2.34)	1.29 (1.84)	0.94 (1.71)
Number censored			
Death	10658	13712	16386
Emigration	5387	6972	11821
Number of fracture cases (%)	646 (0.88)	1092 (0.91)	955 (0.65)

PYR = person-years, SD = standard deviation

Table B. **Sensitivity analysis: Results of the cohort analysis of hip/femur fractures using Cox-regression model (all inpatient cases)**

	PYR	Number of cases	Cases/ 100,000 PYR	Crude HR	Crude 95% CI	Adjusted* HR	Adjusted * 95% CI
**Risperidone**	114,630	747	651.66	Ref=1	-	Ref=1	-
**Other atypical**	155,682	1400	899.27	1.32	(1.21-1.44)	0.91	(0.83-0.999)
**Typical**	139,200	733	526.58	0.78	(0.7-0.86)	0.99	(0.89-1.1)
Age group							
18-39	144,820	143	98.74	Ref=1	-	Ref=1	-
40-49	76,485	127	166.05	1.69	(1.33-2.14)	1.68	(1.33-2.14)
50-59	55,676	161	289.17	2.97	(2.37-3.72)	2.88	(2.30-3.62)
60-69	42,669	360	843.70	8.71	(7.17-10.57)	7.63	(6.23-9.34)
70-79	53,128	984	1852.13	19.27	(16.15-22.99)	15.12	(12.43-18.39)
≥80	36,733	1105	3008.19	31.50	(26.41-37.56)	23.52	(19.25-28.74)
Index diagnosis of schizophrenia	123,059	278	225.91	0.26	(0.23-0.29)	0.87	(0.70-1.09)
Index diagnosis of dementia	44,425	1089	2451.32	4.85	(4.5-5.24)	1.11	(1.01-1.22)
Any time diagnosis of schizophrenia	159,679	408	255.51	0.26	(0.24-0.29)	1.09	(0.91-1.30)
Any time diagnosis of dementia	81,156	1764	2173.59	6.29	(5.83-6.78)	1.40	(1.26-1.56)
One year before exposure date diagnosed with schizophrenia	67,670	160	236.44	0.32	(0.27-0.37)	1.06	(0.85-1.31)

*Adjusted variables including antipsychotic drug, age-group, Index and any time diagnosis (Schizophrenia, dementia), and one year before exposure date diagnosed as schizophrenia

PYR = person-years, CI = 95% confidence interval, HR = hazard ratio

Table C. **Sensitivity analysis: Results of the cohort analysis of hip/femur fractures using Cox-regression model (inpatient and outpatient cases)**

	PYR	Number of cases	Cases/ 100,000 PYR	Crude HR	Crude 95% CI	Adjusted* HR	Adjusted * 95% CI
**Risperidone**	114413	871	761.28	Ref=1	-	Ref=1	-
**Other atypical**	155326	1642	1057.13	1.33	(1.22-1.44)	0.92	(0.85-1.003)
**Typical**	138936	903	649.94	0.82	(0.75-0.9)	1.04	(0.94-1.15)
**Age group**							
18-39	144720	201	138.89	Ref=1	-	Ref=1	-
40-49	76405	162	212.03	1.53	(1.24-1.88)	1.52	(1.24-1.87)
50-59	55609	195	350.66	2.55	(2.1-3.11)	2.46	(2.01-3)
60-69	42545	425	998.94	7.3	(6.17-8.64)	6.31	(5.29-7.53)
70-79	52891	1147	2168.61	15.95	(13.72-18.55)	12.27	(10.36-14.54)
≥80	36506	1286	3522.71	26.01	(22.38-30.23)	19.04	(16-22.66)
Index diagnosis of schizophrenia	122927	336	273.33	0.26	(0.23-0.3)	0.84	(0.69-1.02)
Index diagnosis of dementia	44159	1275	2887.29	4.76	(4.44-5.11)	1.12	(1.03-1.23)
Any time diagnosis of schizophrenia	159484	497	311.63	0.27	(0.25-0.3)	1.1	(0.94-1.3)
Any time diagnosis of dementia	80713	2061	2553.49	6.06	(5.66-6.5)	1.41	(1.28-1.56)
One year before exposure date diagnosed with schizophrenia	67586	193	285.56	0.32	(0.28-0.37)	1.05	(0.86-1.27)

*Adjusted variables including antipsychotic drug, age-group, Index and any time diagnosis (Schizophrenia, dementia), and one year before exposure date diagnosed as schizophrenia

PYR = person-years, CI = 95% confidence interval, HR = hazard ratio

Table D. Stratified analyses: results of the cohort analysis of hip/femur fractures$ using a Cox-regression model in patients with an anytime diagnosis of schizophrenia

	PYR	Number of cases	Cases/ 100,000 PYR	Crude HR	Crude 95% CI	Adjusted HR	Adjusted 95% CI
**Adjusted for age, index diagnosis of dementia, any time diagnosis of dementia**							
Risperidone	72427	173	238.86	Ref=1	-	Ref=1	-
Other atypical	39199	106	270.42	1.12	(0.88-1.43)	0.96	(0.75-1.22)
Typical	48062	119	247.60	1.02	(0.81-1.29)	1.24	(0.98-1.57)
Age group							
18-39	85290	67	78.56	Ref=1		Ref=1	
40-49	38370	52	135.52	1.73	(1.2-2.48)	1.69	(1.18-2.43)
50-59	21079	64	303.62	3.92	(2.78-5.52)	3.78	(2.68-5.33)
60-69	9752	78	799.84	10.35	(7.47-14.36)	9.34	(6.66-13.09)
70-79	4046	90	2224.42	29.22	(21.26-40.15)	23.96	(16.74-34.31)
≥80	1151	47	4083.41	53.71	(36.88-78.24)	41.82	(27.12-64.49)
Index diagnosis of dementia	2211	43	1944.82	8.56	(6.23-11.76)	0.86	(0.59-1.24)
Any time diagnosis of dementia	7497	125	1667.33	9.35	(7.56-11.57)	1.59	(1.19-2.12)
**Adjusted for age**							
Risperidone	72427	173	238.86	Ref=1	-	Ref=1	-
Other atypical	39199	106	270.42	1.12	(0.88-1.43)	0.964	(0.755-1.230)
Typical	48062	119	247.60	1.02	(0.81-1.29)	1.239	(0.979-1.569)
Age group							
18-39	85290	67	78.56	Ref=1		Ref=1	
40-49	38370	52	135.52	1.73	(1.2-2.48)	1.700	(1.183-2.443)
50-59	21079	64	303.62	3.92	(2.78-5.52)	3.892	(2.763-5.484)
60-69	9752	78	799.84	10.35	(7.47-14.36)	10.488	(7.562-14.545)
70-79	4046	90	2224.42	29.22	(21.26-40.15)	30.059	(21.846-41.359)
≥80	1151	47	4083.41	53.71	(36.88-78.24)	55.394	(37.934-80.891)
**Adjusted for index diagnosis of dementia**							

Risperidone	72427	173	238.86	Ref=1	-	Ref=1	-
Other atypical	39199	106	270.42	1.12	(0.88-1.43)	1.061	(0.833-1.353)
Typical	48062	119	247.60	1.02	(0.81-1.29)	1.093	(0.864-1.382)
Index diagnosis of dementia	2211	43	1944.82	8.56	(6.23-11.76)	8.623	(6.256-11.886)
**Adjusted for any time diagnosis of dementia**							
Risperidone	72427	173	238.86	Ref=1	-	Ref=1	-
Other atypical	39199	106	270.42	1.12	(0.88-1.43)	1.048	(0.822-1.335)
Typical	48062	119	247.60	1.02	(0.81-1.29)	1.142	(0.903-1.444)
Any time diagnosis of dementia	7497	125	1667.33	9.35	(7.56-11.57)	9.447	(7.624-11.705)

$ inpatient cases with hip/femur fracture procedure and X-ray within 4 weeks of the diagnosis. PYR = person-years, CI = 95% confidence interval, HR = hazard ratio

Table E. Stratified analyses: results of the cohort analysis of hip/femur fractures[$] using a Cox-regression model in patients without an anytime diagnosis of schizophrenia

	PYR	Number of cases	Cases/ 100,000 PYR	Crude HR	Crude 95% CI	Adjusted HR	Adjusted 95% CI
**Adjusted for age, index diagnosis of dementia, any time diagnosis of dementia**							
Risperidone	42213	557	1319.50	Ref=1	-	Ref=1	-
Other atypical	116502	1273	1092.69	0.83	(0.75-0.91)	0.89	(0.81-0.99)
Typical	91150	604	662.64	0.5	(0.45-0.56)	0.94	(0.83-1.06)
Age group							
18-39	59532	73	122.62	Ref=1		Ref=1	
40-49	38117	73	191.52	1.56	(1.13-2.16)	1.55	(1.12-2.15)
50-59	34600	93	268.79	2.19	(1.61-2.98)	2.11	(1.55-2.87)
60-69	32923	275	835.28	6.81	(5.26-8.81)	5.88	(4.53-7.63)
70-79	49103	883	1798.26	14.71	(11.59-18.68)	11.4	(8.9-14.61)
≥80	35591	1037	2913.66	24.01	(18.94-30.46)	17.67	(13.76-22.69)
Index diagnosis of dementia	42223	1031	2441.80	3.6	(3.32-3.9)	1.15	(1.04-1.27)
Any time diagnosis of dementia	73673	1610	2185.33	4.67	(4.29-5.08)	1.36	(1.21-1.52)
**Adjusted for age**							
Risperidone	42213	557	1319.50	Ref=1	-	Ref=1	-
Other atypical	116502	1273	1092.69	0.83	(0.75-0.91)	0.870	(0.788-0.962)
Typical	91150	604	662.64	0.5	(0.45-0.56)	0.822	(0.731-0.923)
Age group							
18-39	59532	73	122.62	Ref=1		Ref=1	
40-49	38117	73	191.52	1.56	(1.13-2.16)	1.573	(1.137-2.176)
50-59	34600	93	268.79	2.19	(1.61-2.98)	2.202	(1.621-2.992)
60-69	32923	275	835.28	6.81	(5.26-8.81)	6.763	(5.224-8.754)
70-79	49103	883	1798.26	14.71	(11.59-18.68)	14.446	(11.373-18.350)
≥80	35591	1037	2913.66	24.01	(18.94-30.46)	23.507	(18.517-29.840)
**Adjusted for index diagnosis of dementia**							

Risperidone	42213	557	1319.50	Ref=1	-	Ref=1	-
Other atypical	116502	1273	1092.69	0.83	(0.75-0.91)	0.900	(0.815-0.995)
Typical	91150	604	662.64	0.5	(0.45-0.56)	0.759	(0.672-0.857)
Index diagnosis of dementia	42223	1031	2441.80	3.6	(3.32-3.9)	3.368	(3.091-3.669)
**Adjusted for any time diagnosis of dementia**							
Risperidone	42213	557	1319.50	Ref=1	-	Ref=1	-
Other atypical	116502	1273	1092.69	0.83	(0.75-0.91)	0.939	(0.849-1.037)
Typical	91150	604	662.64	0.5	(0.45-0.56)	0.866	(0.768-0.976)
Any time diagnosis of dementia	73673	1610	2185.33	4.67	(4.29-5.08)	4.533	(4.152-4.949)

$ inpatient cases with hip/femur fracture procedure and X-ray within 4 weeks of the diagnosis. PYR = person-years, CI = 95% confidence interval, HR = hazard ratio

Table F. Stratified analyses: results of the cohort analysis of hip/femur fractures[$] using a Cox-regression model in patients aged <80 years

	PYR	Number of cases	Cases/ 100,000 PYR	Crude HR	Crude 95% CI	Adjusted HR	Adjusted 95% CI
**Adjusted for index diagnosis of schizophrenia, index diagnosis of dementia, any time diagnosis of schizophrenia, any time diagnosis of dementia, one year before exposure date diagnosed with schizophrenia**							
Risperidone	106699	476	446.11	Ref=1	-	Ref=1	-
Other atypical	134096	751	560.05	1.23	(1.1-1.38)	0.93	(0.82-1.04)
Typical	132016	521	394.65	0.87	(0.77-0.99)	1	(0.88-1.14)
Index diagnosis of schizophrenia	122513	248	202.43	0.33	(0.29-0.38)	0.81	(0.64-1.04)
Index diagnosis of dementia	26376	496	1880.50	5.18	(4.66-5.75)	1.29	(1.13-1.48)
Any time diagnosis of schizophrenia	158537	351	221.40	0.33	(0.29-0.37)	0.6	(0.5-0.74)
Any time diagnosis of dementia	53752	889	1653.89	6.15	(5.6-6.76)	4.52	(3.99-5.11)
One year before exposure date diagnosed with schizophrenia	67448	145	214.98	0.41	(0.35-0.49)	1.06	(0.84-1.34)
**Adjusted for index diagnosis of schizophrenia**							
Risperidone	106699	476	446.11	Ref=1	-	Ref=1	-
Other atypical	134096	751	560.05	1.23	(1.1-1.38)	0.926	(0.823-1.043)
Typical	132016	521	394.65	0.87	(0.77-0.99)	0.687	(0.606-0.780)
Index diagnosis of schizophrenia	122513	248	202.43	0.33	(0.29-0.38)	0.316	(0.275-0.364)
Adjusted for index diagnosis of schizophrenia,							
Risperidone	106699	476	446.11	Ref=1	-	Ref=1	-
Other atypical	134096	751	560.05	1.23	(1.1-1.38)	0.926	(0.823-1.043)
Typical	132016	521	394.65	0.87	(0.77-0.99)	0.687	(0.606-0.780)
Index diagnosis of schizophrenia	122513	248	202.43	0.33	(0.29-0.38)	0.316	(0.275-0.364)
**Adjusted for index diagnosis of dementia**							
Risperidone	106699	476	446.11	Ref=1	-	Ref=1	-
Other atypical	134096	751	560.05	1.23	(1.1-1.38)	1.119	(0.997-1.257)
Typical	132016	521	394.65	0.87	(0.77-0.99)	1.082	(0.953-1.228)
Index diagnosis of dementia	26376	496	1880.50	5.18	(4.66-5.75)	5.149	(4.616-5.744)

**Adjusted for any time diagnosis of schizophrenia**							
Risperidone	106699	476	446.11	Ref=1	-	Ref=1	-
Other atypical	134096	751	560.05	1.23	(1.1-1.38)	0.843	(0.747-0.950)
Typical	132016	521	394.65	0.87	(0.77-0.99)	0.634	(0.558-0.721)
Any time diagnosis of schizophrenia	158537	351	221.40	0.33	(0.29-0.37)	0.306	(0.270-0.346)
Risperidone	106699	476	446.11	Ref=1	-	Ref=1	-
Other atypical	134096	751	560.05	1.23	(1.1-1.38)	1.075	(0.958-1.207)
Typical	132016	521	394.65	0.87	(0.77-0.99)	1.111	(0.980-1.260)
Any time diagnosis of dementia	53752	889	1653.89	6.15	(5.6-6.76)	6.200	(5.628-6.830)
**Adjusted for one year before exposure date diagnosed with schizophrenia**							
Risperidone	106699	476	446.11	Ref=1	-	Ref=1	-
Other atypical	134096	751	560.05	1.23	(1.1-1.38)	1.109	(0.987-1.245)
Typical	132016	521	394.65	0.87	(0.77-0.99)	0.793	(0.700-0.899)
One year before exposure date diagnosed with schizophrenia	67448	145	214.98	0.41	(0.35-0.49)	0.409	(0.344-0.487)
**Adjusted for any time diagnosis of schizophrenia, any time diagnosis of dementia**							
Risperidone	106699	476	446.11	Ref=1	-	Ref=1	-
Other atypical	134096	751	560.05	1.23	(1.1-1.38)	0.929	(0.825-1.045)
Typical	132016	521	394.65	0.87	(0.77-0.99)	0.971	(0.853-1.105)
Any time diagnosis of schizophrenia	158537	351	221.40	0.33	(0.29-0.37)	0.528	(0.463-0.601)
Any time diagnosis of dementia	53752	889	1653.89	6.15	(5.6-6.76)	5.134	(4.633-5.689)
**Adjusted for index diagnosis of schizophrenia, index diagnosis of dementia, one year before exposure date diagnosed with schizophrenia**							
Risperidone	106699	476	446.11	Ref=1	-	Ref=1	-
Other atypical	134096	751	560.05	1.23	(1.1-1.38)	0.947	(0.842-1.065)
Typical	132016	521	394.65	0.87	(0.77-0.99)	0.917	(0.805-1.044)
Index diagnosis of schizophrenia	122513	248	202.43	0.33	(0.29-0.38)	0.441	(0.369-0.526)
Index diagnosis of dementia	26376	496	1880.50	5.18	(4.66-5.75)	4.159	(3.717-4.654)
One year before exposure date diagnosed with schizophrenia	67448	145	214.98	0.41	(0.35-0.49)	0.926	(0.745-1.152)

$ inpatient cases with hip/femur fracture procedure and X-ray within 4 weeks of the diagnosis. PYR = person-years, CI = 95% confidence interval, HR = hazard ratio

Table G. Stratified analyses: results of the cohort analysis of hip/femur fractures[$] using a Cox-regression model in patients aged 80 years and older

	PYR	Number of cases	Cases/ 100,000 PYR	Crude HR	Crude 95% CI	Adjusted HR	Adjusted 95% CI
**Adjusted for index diagnosis of schizophrenia, index diagnosis of dementia, any time diagnosis of schizophrenia, any time diagnosis of dementia, one year before exposure date diagnosed with schizophrenia**							
Risperidone	7941	254	3198.59	Ref=1	-	Ref=1	-
Other atypical	21605	628	2906.73	0.91	(0.79-1.05)	0.93	(0.8-1.08)
Typical	7197	202	2806.73	0.88	(0.73-1.06)	0.95	(0.79-1.15)
Index diagnosis of schizophrenia	556	20	3597.12	1.24	(0.8-1.93)	0.86	(0.48-1.54)
Index diagnosis of dementia	18058	578	3200.80	1.18	(1.05-1.33)	1.12	(0.97-1.3)
Any time diagnosis of schizophrenia	1151	47	4083.41	1.42	(1.06-1.91)	1.48	(1.01-2.16)
Any time diagnosis of dementia	27418	846	3085.56	1.21	(1.05-1.4)	1.13	(0.95-1.34)
One year before exposure date diagnosed with schizophrenia	228	10	4385.96	1.51	(0.81-2.81)	1.19	(0.58-2.46)
**Adjusted for index diagnosis of schizophrenia**							
Risperidone	7941	254	3198.59	Ref=1	-	Ref=1	-
Other atypical	21605	628	2906.73	0.91	(0.79-1.05)	0.915	(0.791-1.059)
Typical	7197	202	2806.73	0.88	(0.73-1.06)	0.880	(0.732-1.059)
Index diagnosis of schizophrenia	556	20	3597.12	1.24	(0.8-1.93)	1.218	(0.782-1.899)
**Adjusted for index diagnosis of dementia**							
Risperidone	7941	254	3198.59	Ref=1	-	Ref=1	-
Other atypical	21605	628	2906.73	0.91	(0.79-1.05)	0.917	(0.792-1.061)
Typical	7197	202	2806.73	0.88	(0.73-1.06)	0.928	(0.768-1.122)
Index diagnosis of dementia	18058	578	3200.80	1.18	(1.05-1.33)	1.176	(1.040-1.330)
**Adjusted for any time diagnosis of schizophrenia**							
Risperidone	7941	254	3198.59	Ref=1	-	Ref=1	-
Other atypical	21605	628	2906.73	0.91	(0.79-1.05)	0.922	(0.796-1.067)
Typical	7197	202	2806.73	0.88	(0.73-1.06)	0.885	(0.736-1.065)

Any time diagnosis of schizophrenia	1151	47	4083.41	1.42	(1.06-1.91)	1.403	(1.046-1.883)
**Adjusted for any time diagnosis of dementia**							
Risperidone	7941	254	3198.59	Ref=1	-	Ref=1	-
Other atypical	21605	628	2906.73	0.91	(0.79-1.05)	0.919	(0.794-1.063)
Typical	7197	202	2806.73	0.88	(0.73-1.06)	0.923	(0.765-1.115)
Any time diagnosis of dementia	27418	846	3085.56	1.21	(1.05-1.4)	1.205	(1.040-1.397)
**Adjusted for one year before exposure date diagnosed with schizophrenia**							
Risperidone	7941	254	3198.59	Ref=1	-	Ref=1	-
Other atypical	21605	628	2906.73	0.91	(0.79-1.05)	0.914	(0.790-1.058)
Typical	7197	202	2806.73	0.88	(0.73-1.06)	0.881	(0.732-1.059)
One year before exposure date diagnosed with schizophrenia	228	10	4385.96	1.51	(0.81-2.81)	1.482	(0.794-2.764)
**Adjusted for any time diagnosis of schizophrenia, ny time diagnosis of dementia**							
Risperidone	7941	254	3198.59	Ref=1	-	Ref=1	-
Other atypical	21605	628	2906.73	0.91	(0.79-1.05)	0.929	(0.803-1.076)
Typical	7197	202	2806.73	0.88	(0.73-1.06)	0.932	(0.771-1.125)
Any time diagnosis of schizophrenia	1151	47	4083.41	1.42	(1.06-1.91)	1.419	(1.058-1.905)
Any time diagnosis of dementia	27418	846	3085.56	1.21	(1.05-1.4)	1.210	(1.044-1.403)
**Adjusted for index diagnosis of schizophrenia, index diagnosis of dementia, one year before exposure date diagnosed with schizophrenia**							
Risperidone	7941	254	3198.59	Ref=1	-	Ref=1	-
Other atypical	21605	628	2906.73	0.91	(0.79-1.05)	0.922	(0.797-1.068)
Typical	7197	202	2806.73	0.88	(0.73-1.06)	0.934	(0.773-1.130)
Index diagnosis of schizophrenia	556	20	3597.12	1.24	(0.8-1.93)	1.153	(0.687-1.934)
Index diagnosis of dementia	18058	578	3200.80	1.18	(1.05-1.33)	1.184	(1.046-1.341)
One year before exposure date diagnosed with schizophrenia	228	10	4385.96	1.51	(0.81-2.81)	1.417	(0.686-2.925)

$ inpatient cases with hip/femur fracture procedure and X-ray within 4 weeks of the diagnosis. PYR = person-years, CI = 95% confidence interval, HR = hazard ratio

Table H. Stratified analyses: results of the cohort analysis of hip/femur fractures[$] using a Cox-regression model in patients aged less than 80 years and with an any time diagnosis of schizophrenia

	PYR	Number of cases	Cases/ 100,000 PYR	Crude HR	Crude 95% CI	Adjusted HR	Adjusted 95% CI
**Adjusted for index diagnosis of dementia, any time diagnosis of dementia**							
Risperidone	72011	152	211.08	Ref=1	-	Ref=1	-
Other atypical	38681	84	217.16	1.03	(0.79-1.34)	0.99	(0.76-1.29)
Typical	47845	115	240.36	1.14	(0.89-1.45)	1.26	(0.98-1.6)
Index diagnosis of dementia	1796	26	1447.66	7	(4.69-10.45)	1.19	(0.75-1.87)
Any time diagnosis of dementia	6685	91	1361.26	8.06	(6.34-10.24)	7.89	(6.01-10.36)
**Adjusted for index diagnosis of dementia**							
Risperidone	72011	152	211.08	Ref=1	-	Ref=1	-
Other atypical	38681	84	217.16	1.03	(0.79-1.34)	0.999	(0.765-1.305)
Typical	47845	115	240.36	1.14	(0.89-1.45)	1.193	(0.935-1.522)
Index diagnosis of dementia	1796	26	1447.66	7	(4.69-10.45)	7.253	(4.846-10.854)
**Adjusted Any time diagnosis of dementia**							
Risperidone	72011	152	211.08	Ref=1	-	Ref=1	-
Other atypical	38681	84	217.16	1.03	(0.79-1.34)	0.994	(0.761-1.297)
Typical	47845	115	240.36	1.14	(0.89-1.45)	1.250	(0.979-1.594)
Any time diagnosis of dementia	6685	91	1361.26	8.06	(6.34-10.24)	8.252	(6.484-10.501)

$ inpatient cases with hip/femur fracture procedure and X-ray within 4 weeks of the diagnosis. PYR = person-years, CI = 95% confidence interval, HR = hazard ratio

Table I. Stratified analyses: results of the cohort analysis of hip/femur fractures[$] using a Cox-regression model in patients aged less than 80 years and without an any time diagnosis of schizophrenia

	PYR	Number of cases	Cases/ 100,000 PYR	Crude HR	Crude 95% CI	Adjusted HR	Adjusted 95% CI
**Adjusted for index diagnosis of dementia, any time diagnosis of dementia**							
Risperidone	34688	324	934.04	Ref=1	-	Ref=1	-
Other atypical	95416	667	699.04	0.75	(0.66-0.86)	0.88	(0.77-1.01)
Typical	84171	406	482.35	0.52	(0.45-0.6)	0.91	(0.78-1.06)
Index diagnosis of dementia	24580	470	1912.12	3.92	(3.51-4.38)	1.34	(1.17-1.55)
Any time diagnosis of dementia	47067	798	1695.46	4.73	(4.25-5.26)	3.98	(3.48-4.56)
**Adjusted for index diagnosis of dementia**							
Risperidone	34688	324	934.04	Ref=1	-	Ref=1	-
Other atypical	95416	667	699.04	0.75	(0.66-0.86)	0.835	(0.731-0.954)
Typical	84171	406	482.35	0.52	(0.45-0.6)	0.761	(0.653-0.888)
Index diagnosis of dementia	24580	470	1912.12	3.92	(3.51-4.38)	3.690	(3.279-4.152)
**Adjusted Any time diagnosis of dementia**							
Risperidone	34688	324	934.04	Ref=1	-	Ref=1	-
Other atypical	95416	667	699.04	0.75	(0.66-0.86)	0.880	(0.770-1.005)
Typical	84171	406	482.35	0.52	(0.45-0.6)	0.863	(0.742-1.005)
Any time diagnosis of dementia	47067	798	1695.46	4.73	(4.25-5.26)	4.623	(4.139-5.165)

$ inpatient cases with hip/femur fracture procedure and X-ray within 4 weeks of the diagnosis. PYR = person-years, CI = 95% confidence interval, HR = hazard ratio

Table J. Stratified analyses: results of the cohort analysis of hip/femur fractures[$] using a Cox-regression model in patients aged 80 years and older and with an any time diagnosis of schizophrenia

	PYR	Number of cases	Cases/ 100,000 PYR	Crude HR	Crude 95% CI	Adjusted HR	Adjusted 95% CI
**Adjusted for index diagnosis of dementia, any time diagnosis of dementia**							
Risperidone	415	21	5060.24	Ref=1	-	Ref=1	-
Other atypical	519	22	4238.92	0.85	(0.47-1.55)	0.85	(0.46-1.55)
Typical	217	4	1843.32	0.33	(0.11-0.96)	0.33	(0.11-0.96)
Index diagnosis of dementia	415	17	4096.39	1.04	(0.57-1.88)	0.91	(0.47-1.77)
Any time diagnosis of dementia	812	34	4187.19	1.12	(0.59-2.13)	1.16	(0.58-2.33)
**Adjusted for index diagnosis of dementia**							
Risperidone	415	21	5060.24	Ref=1	-	Ref=1	-
Other atypical	519	22	4238.92	0.85	(0.47-1.55)	0.855	(0.468-1.561)
Typical	217	4	1843.32	0.33	(0.11-0.96)	0.329	(0.112-0.963)
Index diagnosis of dementia	415	17	4096.39	1.04	(0.57-1.88)	0.968	(0.527-1.777)
**Adjusted Any time diagnosis of dementia**							
Risperidone	415	21	5060.24	Ref=1	-	Ref=1	-
Other atypical	519	22	4238.92	0.85	(0.47-1.55)	0.843	(0.461-1.539)
Typical	217	4	1843.32	0.33	(0.11-0.96)	0.329	(0.113-0.962)
Any time diagnosis of dementia	812	34	4187.19	1.12	(0.59-2.13)	1.117	(0.587-2.126)

$ inpatient cases with hip/femur fracture procedure and X-ray within 4 weeks of the diagnosis. PYR = person-years, CI = 95% confidence interval, HR = hazard ratio

Table K. Stratified analyses: results of the cohort analysis of hip/femur fractures[$] using a Cox-regression model in patients aged 80 years and older and without an any time diagnosis of schizophrenia

	PYR	Number of cases	Cases/ 100,000 PYR	Crude HR	Crude 95% CI	Adjusted HR	Adjusted 95% CI
**Adjusted for index diagnosis of dementia, any time diagnosis of dementia**							
Risperidone	7526	233	3095.93	Ref=1	-	Ref=1	-
Other atypical	21086	606	2873.94	0.93	(0.8-1.08)	0.94	(0.81-1.09)
Typical	6980	198	2836.68	0.92	(0.76-1.11)	0.995	(0.82-1.21)
Index diagnosis of dementia	17643	561	3179.73	1.2	(1.06-1.35)	1.14	(0.98-1.32)
Any time diagnosis of dementia	26606	812	3051.94	1.22	(1.05-1.42)	1.13	(0.95-1.35)
**Adjusted for index diagnosis of dementia**							
Risperidone	7526	233	3095.93	Ref=1	-	Ref=1	-
Other atypical	21086	606	2873.94	0.93	(0.8-1.08)	0.937	(0.805-1.090)
Typical	6980	198	2836.68	0.92	(0.76-1.11)	0.980	(0.807-1.191)
Index diagnosis of dementia	17643	561	3179.73	1.2	(1.06-1.35)	1.201	(1.058-1.363)
**Adjusted Any time diagnosis of dementia**							
Risperidone	7526	233	3095.93	Ref=1	-	Ref=1	-
Other atypical	21086	606	2873.94	0.93	(0.8-1.08)	0.938	(0.806-1.092)
Typical	6980	198	2836.68	0.92	(0.76-1.11)	0.972	(0.800-1.180)
Any time diagnosis of dementia	26606	812	3051.94	1.22	(1.05-1.42)	1.222	(1.050-1.423)

$ inpatient cases with hip/femur fracture procedure and X-ray within 4 weeks of the diagnosis. PYR = person-years, CI = 95% confidence interval, HR = hazard ratio

Table L. Selection of confounding factors for hip/femur fracture based on minimum of 10% change in the crude estimate (overall) hazard ratio (HR) and 95% confidence interval (CI)

	Other Atypical	Risperidone	Ratio	Typical	Ratio
PYR	155682	114630		139200	
Number of events	1400	747		733	
Events / 100,000 person-years	899.3	651.7		526.6	
Crude	Reference	0.757 (0.692-0.828)	-	0.588 (0.538-0.643)	-
HR adjusted for gender		0.766 (0.700-0.837)	1.012	0.594 (0.543-0.650)	1.010
HR adjusted for age		1.107 (1.013-1.211)	1.462	0.986 (0.901-1.081)	1.677
**Adjusted for Primary indication**					
HR adjusted for Schizophrenia		1.024 (0.935-1.122)	1.353	0.629 (0.575-0.688)	1.070
HR adjusted for Bipolar disorder		0.731 (0.669-0.800)	0.966	0.567 (0.519-0.621)	0.964
HR adjusted for Major depression		0.720 (0.658-0.788)	0.951	0.556 (0.509-0.609)	0.946
HR adjusted for Autism		0.757 (0.693-0.828)	1.000	0.588 (0.538-0.644)	1.000
HR adjusted for Dementia		0.857 (0.784-0.938)	1.132	0.869 (0.791-0.955)	1.478
HR adjusted for Disruptive behavior		0.756 (0.692-0.827)	0.999	0.588 (0.538-0.643)	1.000
**Adjusted for 2000-2012 diagnosis**					
HR adjusted for Schizophrenia		1.127 (1.028-1.236)	1.489	0.641 (0.586-0.702)	1.090
HR adjusted for Bipolar disorder		0.707 (0.646-0.773)	0.934	0.551 (0.504-0.603)	0.937
HR adjusted for Major depression		0.701 (0.641-0.767)	0.926	0.544 (0.497-0.595)	0.925
HR adjusted for Autism		0.759 (0.694-0.829)	1.003	0.589 (0.538-0.644)	1.002
HR adjusted for Dementia		0.904 (0.826-0.988)	1.194	0.950 (0.866-1.042)	1.616
HR adjusted for Disruptive behavior		0.757 (0.692-0.828)	1.000	0.588 (0.538-0.643)	1.000
**Adjusted for diagnosis 12 months prior to the index exposure**					
HR adjusted for Schizophrenia		0.851 (0.778-0.931)	1.124	0.602 (0.550-0.658)	1.024
HR adjusted for Bipolar disorder		0.746 (0.682-0.815)	0.985	0.580 (0.530-0.634)	0.986
HR adjusted for Major depression		0.724 (0.662-0.792)	0.956	0.562 (0.514-0.615)	0.956
HR adjusted for Autism		0.758 (0.693-0.828)	1.001	0.589 (0.538-0.644)	1.002
HR adjusted for Dementia		0.796 (0.728-0.870)	1.052	0.662 (0.605-0.725)	1.126
HR adjusted for Disruptive behavior		0.757 (0.692-0.827)	1.000	0.588 (0.538-0.643)	1.000
HR adjusted for Seizures/Epilepsies		0.758 (0.693-0.828)	1.001	0.589 (0.538-0.644)	1.002
HR adjusted for Renal dysfunction		0.764 (0.699-0.835)	1.009	0.593 (0.543-0.649)	1.009
HR adjusted for Other organic psychiatric disorder		0.755 (0.691-0.826)	0.997	0.591 (0.541-0.646)	1.005
HR adjusted for Other psychosis		0.761 (0.696-0.833)	1.005	0.587 (0.537-0.642)	0.998
HR adjusted for Neurotic stress related or somatoform disorder		0.728 (0.666-0.797)	0.962	0.569 (0.520-0.622)	0.968
**Adjusted for possible bone-related medications 12 months prior to the index exposure**					
HR adjusted for glucorticosteroids		0.765 (0.699-0.836)	1.011	0.590 (0.540-0.645)	1.003
HR adjusted for anxiolytics, sedatives and hypnotics		0.745 (0.681-0.815)	0.984	0.578 (0.528-0.633)	0.983
HR adjusted for antidepressants		0.716 (0.654-0.784)	0.946	0.554 (0.505-0.606)	0.942
HR adjusted for proton-pump inhibitors		0.766 (0.700-0.837)	1.012	0.594 (0.543-0.649)	1.010
HR adjusted for hormone replacement therapy		0.757 (0.692-0.828)	1.000	0.588 (0.538-0.643)	1.000
HR adjusted for oral contraceptives		0.754 (0.689-0.824)	0.996	0.588 (0.538-0.643)	1.000
HR adjusted for calcium supplements		0.758 (0.693-0.828)	1.001	0.584 (0.534-0.638)	0.993
HR adjusted for thiazide diuretics		0.777 (0.710-0.849)	1.026	0.604 (0.552-0.661)	1.027
HR adjusted for gastrointestinal medications		0.790 (0.722-0.864)	1.044	0.598 (0.547-0.654)	1.017
HR adjusted for opiates		0.779 (0.712-0.852)	1.029	0.602 (0.551-0.659)	1.024
HR adjusted for antihypertensive medications		0.760 (0.695-0.831)	1.004	0.587 (0.537-0.642)	0.998
HR adjusted for glucorticosteroids ≥ 90 days		0.762 (0.697-0.833)	1.007	0.590 (0.539-0.645)	1.003

Table M. Selection of confounding factors for non-hip/femur fracture based on minimum of 10% change in the crude estimate (overall) hazard ratio (HR) and 95% confidence interval (CI)

	Other Atypical	Risperidone	Ratio	Typical	Ratio
PYR	155847	114504		138576	
Number of events	1092	646		955	
Events / 100,000 person-years	700.7	564.2		689.2	
Crude	Reference	0.823 (0.746-0.907)	-	0.985 (0.903-1.075)	-
HR adjusted for gender		0.835 (0.757-0.920)	1.015	0.997 (0.914-1.088)	1.012
HR adjusted for age		0.880 (0.798-0.972)	1.069	1.067 (0.976-1.165)	1.083
**Adjusted for Primary indication**					
HR adjusted for Schizophrenia		0.919 (0.831-1.017)	1.117	1.012 (0.928-1.104)	1.027
HR adjusted for Bipolar disorder		0.818 (0.742-0.902)	0.994	0.979 (0.898-1.069)	0.994
HR adjusted for Major depression		0.848 (0.768-0.936)	1.030	1.019 (0.933-1.113)	1.035
HR adjusted for Autism		0.822 (0.745-0.906)	0.999	0.985 (0.903-1.074)	1.000
HR adjusted for Dementia		0.840 (0.762-0.927)	1.021	1.044 (0.955-1.142)	1.060
HR adjusted for Disruptive behavior		0.822 (0.746-0.907)	0.999	0.985 (0.903-1.075)	1.000
**Adjusted for 2000-2012 diagnosis**					
HR adjusted for Schizophrenia		0.911 (0.823-1.009)	1.107	1.011 (0.926-1.103)	1.026
HR adjusted for Bipolar disorder		0.845 (0.766-0.932)	1.027	1.010 (0.926-1.103)	1.025
HR adjusted for Major depression		0.877 (0.794-0.968)	1.066	1.052 (0.963-1.150)	1.068
HR adjusted for Autism		0.822 (0.745-0.906)	0.999	0.985 (0.903-1.074)	1.000
HR adjusted for Dementia		0.857 (0.777-0.946)	1.041	1.086 (0.993-1.187)	1.103
HR adjusted for Disruptive behavior		0.823 (0.746-0.907)	1.000	0.985 (0.903-1.075)	1.000
**Adjusted for diagnosis 12 months prior to the index exposure**					
HR adjusted for Schizophrenia		0.858 (0.777-0.947)	1.043	0.994 (0.911-1.084)	1.009
HR adjusted for Bipolar disorder		0.825 (0.748-0.910)	1.002	0.988 (0.906-1.078)	1.003
HR adjusted for Major depression		0.845 (0.766-0.933)	1.027	1.014 (0.928 1.107)	1.029
HR adjusted for Autism		0.823 (0.746-0.907)	1.000	0.985 (0.903-1.075)	1.000
HR adjusted for Dementia		0.829 (0.752-0.915)	1.007	1.004 (0.919-1.096)	1.019
HR adjusted for Disruptive behavior		0.823 (0.746-0.907)	1.000	0.985 (0.903-1.075)	1.000
HR adjusted for Seizures/Epilepsies		0.821 (0.745-0.905)	0.998	0.984 (0.902-1.074)	0.999
HR adjusted for Renal dysfunction		0.824 (0.748-0.909)	1.001	0.987 (0.905-1.077)	1.002
HR adjusted for Other organic psychiatric disorder		0.822 (0.745-0.906)	0.999	0.988 (0.905-1.077)	1.003
HR adjusted for Other psychosis		0.818 (0.742-0.902)	0.994	0.987 (0.905-1.077)	1.002
HR adjusted for Neurotic stress related or somatoform disorder		0.855 (0.774-0.944)	1.039	1.019 (0.933-1.113)	1.035
**Adjusted for possible bone-related medications 12 months prior to the index exposure**					
HR adjusted for glucorticosteroids		0.827 (0.750-0.912)	1.005	0.987 (0.905-1.076)	1.002
HR adjusted for anxiolytics, sedatives and hypnotics		0.862 (0.781-0.952)	1.047	1.042 (0.954-1.138)	1.058
HR adjusted for antidepressants		0.871 (0.789-0.962)	1.058	1.051 (0.961-1.149)	1.067
HR adjusted for proton-pump inhibitors		0.826 (0.749-0.911)	1.004	0.988 (0.906-1.078)	1.003
HR adjusted for hormone replacement therapy		0.824 (0.748-0.909)	1.001	0.985 (0.903-1.074)	1.000
HR adjusted for oral contraceptives		0.827 (0.750-0.912)	1.005	0.985 (0.903-1.075)	1.000
HR adjusted for calcium supplements		0.824 (0.747-0.908)	1.001	0.980 (0.898-1.069)	0.995
HR adjusted for thiazide diuretics		0.826 (0.749-0.911)	1.004	0.990 (0.907-1.080)	1.005
HR adjusted for gastrointestinal medications		0.840 (0.761-0.926)	1.021	0.994 (0.911-1.084)	1.009
HR adjusted for opiates		0.832 (0.754-0.917)	1.011	0.994 (0.911-1.085)	1.009
HR adjusted for antihypertensive medications		0.824 (0.748-0.909)	1.001	0.984 (0.903-1.074)	0.999
HR adjusted for glucorticosteroids ≥ 90 days		0.826 (0.749-0.911)	1.004	0.986 (0.904-1.076)	1.001

Table N. Description of cases and matched controls with respect to number of selected controls.

	Current period		Recent period		Past period	
	**Cases n=2535 number (%)**	**Controls n=10,140 number (%)**	**Cases n= 2460 number (%)**	**Controls n= 9840 number (%)**	**Cases n=1334 number (%)**	**Controls n= 5336 number (%)**
**Age (years), mean±SD**	76.14±13.43	75.71±13.33	76.18±13.59	75.78±13.50	74.47±14.42	74.21±14.39
**Age group**						
18-39	86 (3.4)	350 (3.5)	90 (3.7)	365 (3.7)	59 (4.4)	239 (4.5)
40-49	72 (2.8)	312 (3.1)	64 (2.6)	259 (2.6)	48 (3.6)	223 (4.2)
50-59	113 (4.5)	451 (4.5)	107 (4.4)	488 (5.0)	86 (6.5)	328 (6.2)
60-69	220 (8.7)	965 (9.5)	221 (9.0)	882 (9.0)	121 (9.1)	481 (9.0)
≥70	2044 (80.6)	8062 (79.5)	1978 (80.4)	7846 (79.7)	1020 (76.5)	4065 (76.2)
**Gender**						
Male	1121 (44.2)	4484 (44.2)	1080 (43.9)	4320 (43.9)	589 (44.2)	2356 (44.2)
Female	1414 (55.8)	5656 (55.8)	1380 (56.1)	5520 (56.1)	745 (55.9)	2980 (55.9)
**Follow up period (days), mean±SD**	732.37±606.59	731.96±606.88	696.55±681.20	695.81±681.34	792.25±782.35	791.76±782.55
**Index year**						
2001	247 (9.7)	870 (8.6)	240 (9.8)	854 (8.7)	159 (11.9)	548 (10.3)
2002	246 (9.7)	1033 (10.2)	233 (9.5)	975 (9.9)	153 (11.5)	635 (11.9)
2003	269 (10.6)	1147 (11.3)	269 (10.9)	1148 (11.7)	185 (13.9)	759 (14.2)
2004	277 (10.9)	1076 (10.6)	286 (11.6)	1119 (11.4)	197 (14.8)	832 (15.6)
2005	245 (9.7)	1256 (12.4)	254 (10.3)	970 (9.9)	151 (11.3)	727 (13.6)
2006	265 (10.5)	1331 (13.1)	256 (10.4)	1058 (10.8)	162 (12.1)	590 (11.1)
2007	269 (10.6)	1034 (10.2)	260 (10.6)	1063 (10.8)	148 (11.1)	529 (9.9)
2008	226 (8.9)	672 (6.6)	229 (9.3)	895 (9.1)	105 (7.9)	384 (7.2)
2009	223 (8.8)	663 (6.5)	226 (9.2)	822 (8.4)	52 (3.9)	174 (3.3)
2010	158 (6.2)	546 (5.4)	157 (6.4)	633 (6.4)	13 (0.97)	86 (1.6)
2011	94 (3.7)	399 (3.9)	48 (2.0)	273 (2.8)	7 (0.52)	56 (1.1)
2012.1-2012.6$^{\$}$	16 (0.63)	113 (1.11)	2 (0.08)	30 (0.30)	2 (0.15)	16 (0.30)
**Fracture year**						
2001	6 (0.24)	24 (0.24)	2 (0.08)	8 (0.08)	12 (0.90)	48 (0.90)
2002	55 (2.2)	220 (2.2)	39 (1.6)	156 (1.6)	36 (2.7)	144 (2.7)
2003	107 (4.2	428 (4.2)	79 (3.2)	316 (3.2)	68 (5.1)	272 (5.1)
2004	129 (5.1)	516 (5.1)	129 (5.2)	516 (5.2)	100 (7.5)	400 (7.5)
2005	179 (7.1)	716 (7.1)	177 (7.2)	708 (7.2)	130 (9.8)	520 (9.8)
2006	196 (7.7)	784 (7.7)	162 (6.6)	648 (6.6)	163 (12.2)	652 (12.2)
2007	228 (9.0)	912 (9.0)	222 (9.0)	888 (9.0)	190 (14.2)	760 (14.2)

2008	281 (11.1)	1124 (11.1)	267 (10.9)	1068 (10.9)	202 (15.1)	808 (15.1)
2009	341 (13.5)	1364 (13.5)	316 (12.9)	1264 (12.9)	214 (16.0)	856 (16.0)
2010	316 (12.5)	1264 (12.5)	336 (13.7)	1344 (13.7)	219 (16.4)	876 (16.4)
2011	346 (13.7)	1384 (13.7)	381 (15.5)	1524 (15.5)	-	-
2012[%]	351 (13.9)	1404 (13.9)	350 (14.2)	1400 (14.2)	-	-
**Primary indication (diagnosis on index date)**						
Schizophrenia	230 (9.1)	1178 (11.6)	223 (9.1)	1171 (11.9)	170 (12.7)	894 (16.8)
Bipolar disorder	49 (1.9)	302 (3.0)	44 (1.8)	287 (2.9)	25 (1.9)	210 (3.9)
Major depression	142 (5.6)	712 (7.0)	145 (5.9)	732 (7.4)	94 (7.1)	439 (8.2)
Autism	0 (0.00)	1 (0.01)	1 (0.04)	5 (0.05)	0 (0.00)	1 (0.02)
Dementia	1115 (44.0)	4072 (40.2)	1058 (43.0)	3995 (40.6)	511 (38.3)	1947 (36.5)
Disruptive behaviors	1 (0.04)	4 (0.04)	1 (0.04)	3 (0.03)	0 (0.00)	1 (0.02)
**Previous Diagnosis[§]**						
Schizophrenia	349 (13.8)	1636 (16.1)	337 (13.7)	1655 (16.8)	243 (18.2)	1233 (23.1)
Bipolar disorder	163 (6.4)	828 (8.2)	155 (6.3)	829 (8.4)	102 (7.7)	574 (10.8)
Major depression	380 (15.0)	1663 (16.4)	373 (15.2)	1675 (17.0)	228 (17.1)	1049 (19.7)
Autism	3 (0.1)	16 (0.2)	5 (0.2)	21 (0.21)	3 (0.2)	10 (0.19)
Dementia	1721 (67.9)	6344 (62.6)	1634 (66.4)	6193 (62.9)	815 (61.1)	3179 (59.6)
Disruptive behaviors	1 (0.04)	2 (0.02)	1 (0.04)	1 (0.01)	1 (0.07)	3 (0.06)
**2000-2012 Dementia**						
Schizophrenia & Dementia	128 (5.1)	418 (4.1)	118 (4.8)	389 (4.0)	80 (6.0)	284 (5.3)
Bipolar disorder & Dementia	73 (2.88)	332 (3.3)	71 (2.9)	304 (3.1)	43 (3.2)	228 (4.3)
Major depression & Dementia	221 (8.72)	881 (8.7)	201 (8.2)	869 (8.8)	124 (9.3)	550 (10.3)

[$] Year 2012 only included subjects for half a year (from 2012.1 to 2012.6)

[%] Fracture cases in 2012 were included the whole year (from 2012.1 to 2012.12)

[§] Previous diagnosis was defined as: during 2000-2012, subject having the main diagnosis from the Admission data or the same diagnosis three times from Ambulatory data

Table O. Crude and Adjusted Odds Ratio (OR) for hip/femur fracture after antipsychotic exposure among cases versus controls (*post hoc* analysis)

	N of Cases	N of Controls	Crude OR (95% CI)	Adjusted OR$ (95% CI)
**Hip/femur fracture**				
Current period				
Risperidone	742	2892	1 (reference)	1 (reference)
Aripiprazole	56	238	0.915 (0.674-1.241)	0.911 (0.671-1.238)
Clozapine	44	213	0.802 (0.573-1.123)	0.890 (0.633-1.251)
Olanzapine	195	732	1.038 (0.870-1.240)	1.053 (0.881-1.258)
Quetiapine	1431	5810	0.956 (0.862-1.061)	0.916 (0.824-1.018)
Ziprasidone	11	42	1.022 (0.523-1.998)	1.012 (0.516-1.984)
Recent period				
Risperidone	806	3083	1 (reference)	1 (reference)
Aripiprazole	46	215	0.808 (0.578-1.130)	0.816 (0.583-1.142)
Clozapine	35	203	0.653 (0.450-0.946)	0.719 (0.495-1.045)
Olanzapine	168	720	0.892 (0.741-1.074)	0.900 (0.747-1.084)
Quetiapine	1368	5168	1.034 (0.932-1.147)	0.997 (0.897-1.108)
Ziprasidone	16	46	1.338 (0.751-2.384)	1.365 (0.763-2.440)
Past period				
Risperidone	493	1824	1 (reference)	1 (reference)
Aripiprazole	18	74	0.895 (0.528-1.519)	0.884 (0.519-1.508)
Clozapine	25	123	0.749 (0.480-1.170)	0.805 (0.514-1.261)
Olanzapine	106	458	0.855 (0.676-1.081)	0.854 (0.674-1.081)
Quetiapine	586	2246	0.985 (0.854-1.136)	0.919 (0.794-1.064)
Ziprasidone	7	33	0.781 (0.343-1.782)	0.793 (0.346-1.820)

$ Multivariate logistic regression model adjusting for age group, index and any time diagnosis (schizophrenia, dementia), and one year before exposure date diagnosed with schizophrenia
Current period: within 1 year prior to the fracture index date,
Recent period: between 1 year and 3 years prior to the fracture index date,
Past period: between 3 years and 5 years prior to the fracture index date.
OR = odds ratio,
CI = confidence intervals,
N = number of patients

Table P Crude and Adjusted Odds Ratio (OR) for non-hip/femur fracture after antipsychotic exposure among cases versus controls (*post hoc* analysis)

	**N of Cases**	**N of Controls**	**Crude OR (95% CI)**	**Adjusted OR$ (95% CI)**
**Non-hip/femur fracture**				
Current period				
Risperidone	613	2745	1 (reference)	1 (reference)
Aripiprazole	100	428	1.053 (0.827-1.340)	1.046 (0.821-1.332)
Clozapine	47	339	0.616 (0.448-0.848)	0.642 (0.466-0.884)
Olanzapine	260	902	1.294 (1.098-1.525)	1.260 (1.069-1.486)
Quetiapine	1076	3888	1.273 (1.133-1.429)	1.160 (1.026-1.310)
Ziprasidone	29	89	1.467 (0.955-2.255)	1.480 (0.961-2.279)
Recent period				
Risperidone	670	2780	1 (reference)	1 (reference)
Aripiprazole	87	367	0.985 (0.764-1.271)	0.973 (0.754-1.256)
Clozapine	34	321	0.436 (0.302-0.627)	0.478 (0.332-0.690)
Olanzapine	241	858	1.167 (0.988-1.380)	1.132 (0.957-1.339)
Quetiapine	996	3644	1.163 (1.036-1.307)	1.014 (0.898-1.145)
Ziprasidone	25	95	1.096 (0.696-1.728)	1.184 (0.749-1.871)
Past period				
Risperidone	485	1952	1 (reference)	1 (reference)
Aripiprazole	38	149	1.028 (0.705-1.499)	1.078 (0.737-1.577)
Clozapine	27	199	0.543 (0.359-0.823)	0.573 (0.377-0.871)
Olanzapine	155	631	0.988 (0.807-1.210)	0.940 (0.765-1.153)
Quetiapine	487	1724	1.174 (1.011-1.364)	0.971 (0.830-1.135)
Ziprasidone	12	67	0.718 (0.384-1.343)	0.735 (0.391-1.383)

$Multivariate logistic regression model adjusting for index and any time diagnosis schizophrenia
Current period: within 1 year prior to the fracture index date,
Recent period: between 1 year and 3 years prior to the fracture index date,
Past period: between 3 years and 5 years prior to the fracture index date.
OR = odds ratio
CI = confidence intervals
N = number of patients

Table Q. Crude and Adjusted Odds Ratio for antipsychotic exposure among cases versus controls in patients who did not change antipsychotic drug

	Cases	Controls	Crude OR (95% CI)	Adjust OR$ (95% CI)
**Hip/femur fracture**	Number who did not change medication between the current and recent periods			
Risperidone	364	1982	1 (reference)	1 (reference)
Other Atypical	933	4631	1.13 (0.97-1.31)	1.07 (0.92-1.25)
	Number who did not change medication between the current and past periods			
Risperidone	308	1824	1 (reference)	1 (reference)
Other Atypical	748	3713	1.31 (1.11-1.54)	1.17 (0.99-1.39)
**Non-hip/femur fracture**	Number who did not change medication between the current and recent periods			
Risperidone	308	1824	1 (reference)	1 (reference)
Other Atypical	748	3713	1.31 (1.11-1.54)	1.17 (0.99-1.39)
	Number who did not change medication between the current and past periods			
Risperidone	148	958	1 (reference)	1 (reference)
Other Atypical	290	1658	1.13 (0.88-1.45)	1.03 (0.79-1.33)

$ Multivariate logistic regression model adjusted by age group, index and any time diagnosis (schizophrenia, dementia), and one year before exposure date diagnosed with schizophrenia for hip/femur fracture, and by index and any time diagnosis of schizophrenia for non-hip/femur fractures
OR = odds ratio, 95% CI = 95% confidence intervals
