# Supplementary figures and images for: The risk of bone fracture after long-term risperidone exposure is not increased compared to other atypical antipsychotics: A retrospective cohort study
Source: PLoS One. 2019 Sep 5;14(9):e0221948. doi: 10.1371/journal.pone.0221948 (PMC6728018; doi:10.1371/journal.pone.0221948)

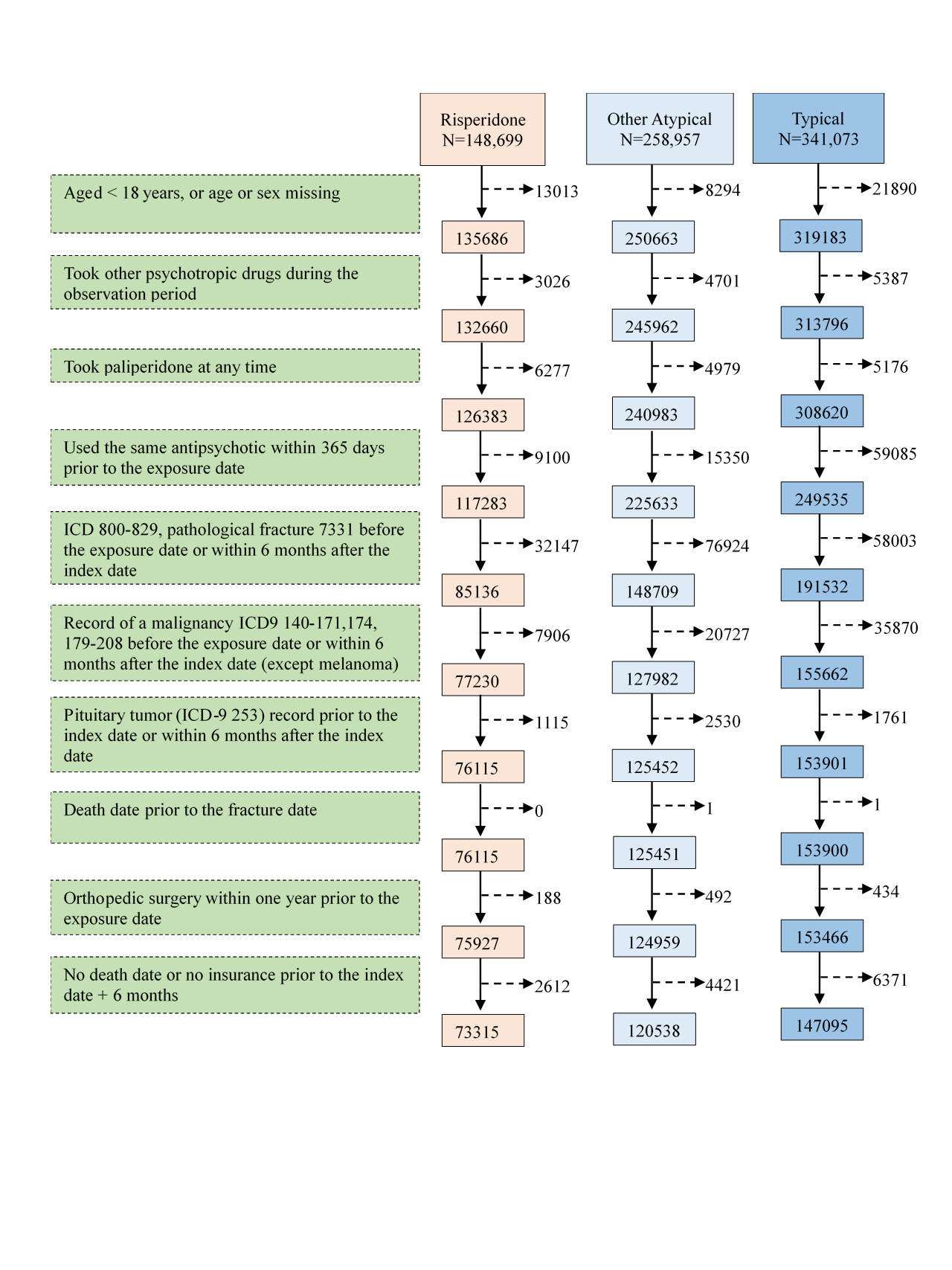

Supplement: S1 Fig — (TIF) [file pone.0221948.s002.tif]
